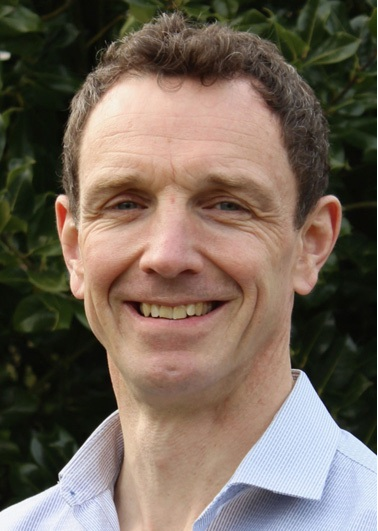# Generation and Reactivity of C(1)‐Ammonium Enolates by Using Isothiourea Catalysis

**DOI:** 10.1002/chem.202002059

**Published:** 2020-11-10

**Authors:** Calum McLaughlin, Andrew D. Smith

**Affiliations:** ^1^ EaStCHEM School of Chemistry University of St Andrews North Haugh Fife KY16 9ST Scotland

**Keywords:** aryloxides, C(1)-ammonium enolates, catalyst turnover, formal cycloaddition, isothioureas

## Abstract

C(1)‐Ammonium enolates are powerful, catalytically generated synthetic intermediates applied in the enantioselective α‐functionalisation of carboxylic acid derivatives. This minireview describes the recent developments in the generation and application of C(1)‐ammonium enolates from various precursors (carboxylic acids, anhydrides, acyl imidazoles, aryl esters, α‐diazoketones, alkyl halides) using isothiourea Lewis base organocatalysts. Their synthetic utility in intra‐ and intermolecular enantioselective C−C and C−X bond forming processes on reaction with various electrophiles will be showcased utilising two distinct catalyst turnover approaches.

## Graphical abstract

**C(1)‐Ammonium enolates** have found widespread application for the enantioselective α‐functionalisation of carboxylic acid derivatives. This minireview highlights the recent advances in the generation of C(1)‐ammonium enolate intermediates by using isothiourea Lewis base organocatalysts and various precursors. The synthetic utility is showcased in intra‐ and intermolecular enantioselective C−C and C−X bond forming processes on reaction with various electrophiles utilising two distinct catalyst turnover approaches.

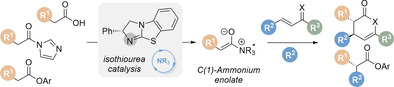

## 1 Introduction

**Figure 1:**
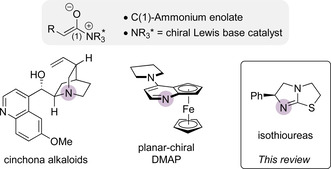
C(1)‐Ammonium enolates.

Synthetic methods that allow the catalytic, stereoselective preparation of enantioenriched compounds is a central research goal in organic synthesis. Lewis base organocatalysis is recognised as an established branch of enantioselective synthesis, enabling the efficient transformation of simple starting materials to complex molecular scaffolds with high diastereo‐ and enantiocontrol.^[1]^ Various activation modes have been accessed through the design of novel Lewis base catalyst architectures such as secondary amines (iminium, enamine, or SOMO catalysis),^[2]^ *N*‐heterocyclic carbenes (acyl azolium, azolium enolate)^[3]^ and tertiary phosphines (β‐phosphonium α‐carbanions),^[4]^ enabling enantioselective functionalisation over a broad substrate range. C(1)‐Ammonium enolate intermediates,^[5]^ derived from chiral tertiary amine Lewis base catalysts such as cinchona alkaloids,^[6]^ planar chiral DMAP derivatives^[7]^ and isothioureas (Figure 1)^[8]^ have emerged as synthetically useful intermediates for the enantioselective synthesis of α‐functionalised carbonyl compounds at the carboxylic acid oxidation level.

**Scheme 1:**
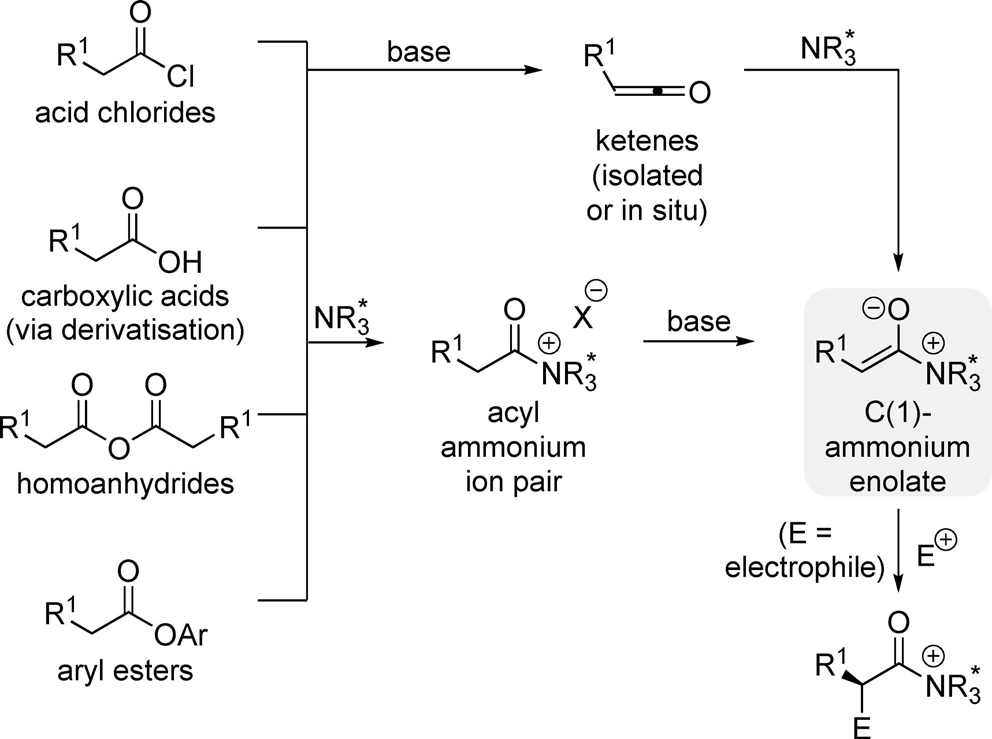
Overview of C(1)‐ammonium enolate generation.

Seminal work by Wynberg demonstrated C(1)‐ammonium enolate intermediates could be accessed by direct nucleophilic addition of a Lewis base catalyst onto a ketene starting material.^[9]^ However, ketenes are typically unstable to long‐term storage and are prone to dimerisation. Alternative methods have focused on the in situ generation of C(1)‐ammonium enolates from bench stable carboxylic acid derivatives. These strategies all rely on the acylation of the Lewis base by a carboxylic acid derivative, followed by deprotonation of the subsequent acyl ammonium ion to give the C(1)‐ammonium enolate intermediate (Scheme 1). Carboxylic acid chlorides (through in situ ketene formation),^[10]^ carboxylic acids (via derivatisation to an activated ester,^[11]^ or mixed anhydride),^[12]^ homoanhydrides^[13]^ and electron deficient aryl esters have been shown to be effective bench stable starting materials for C(1)‐ammonium enolate generation.^[14]^ The nucleophilic enolate can engage in stereoselective C−C and C−X bond formation on reaction with an electrophile, giving α‐functionalised carbonyl compounds at the carboxylic acid oxidation level following catalyst turnover. Owing to the mild basic reaction conditions, enolisable tertiary stereogenic centres can be formed in high enantiopurity with good functional group tolerance. Therefore, tertiary amine catalysis via C(1)‐ammonium enolate intermediates is an attractive approach for the synthesis of complex molecules containing α‐functionalised carboxylic acid, esters and amide functionality, motifs that are found in many biologically relevant molecules.^[15]^

**Scheme 2:**
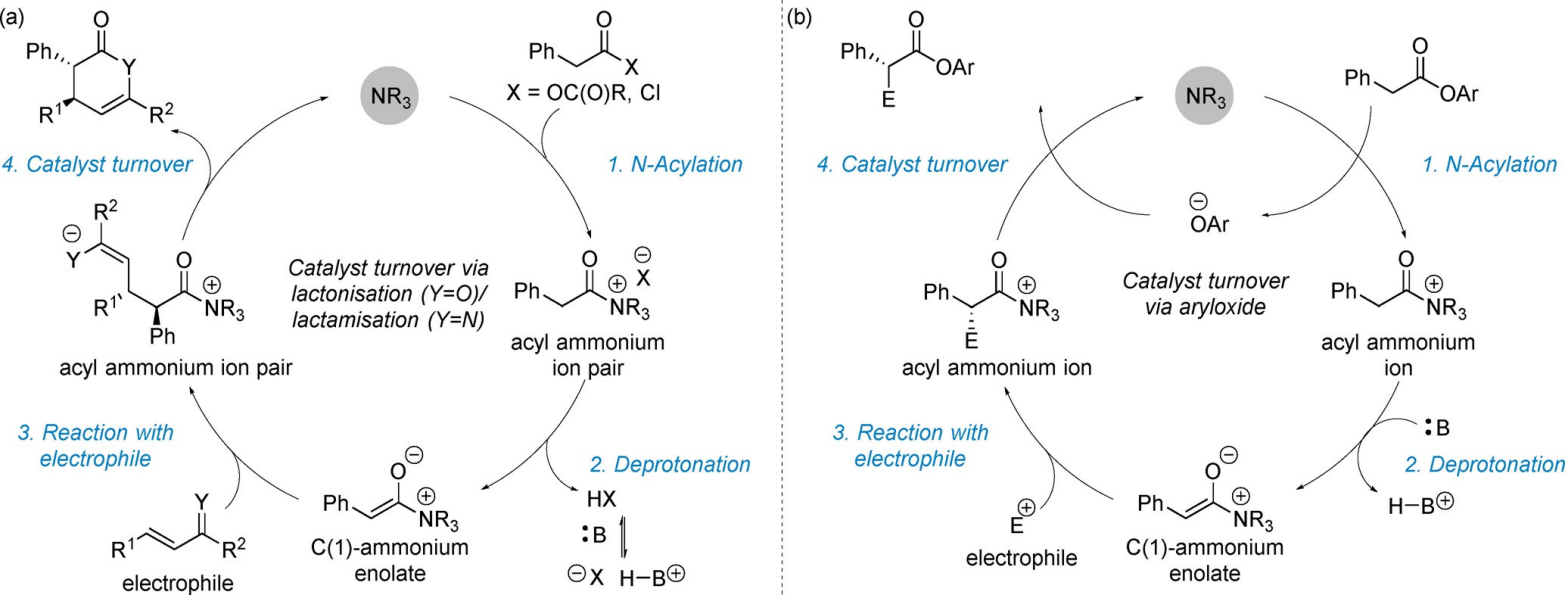
Catalyst turnover in C(1)‐ammonium enolate catalysis (a) via lactonisation (X=O)/lactamisation (X=N) and (b) via aryloxide.

The catalytic reactivity of C(1)‐ammonium enolates is governed by two distinct catalyst turnover approaches (Scheme 2). Traditional strategies effected catalyst turnover via an intramolecular lactonisation/lactamisation approach (Scheme 2 a). Mechanistically, this involves *N*‐acylation of the Lewis base by the carboxylic acid derivative (anhydride, ketene) to give an acyl ammonium ion pair. The C(2)‐protons become more acidic due to the electron withdrawing nature of the cationic nitrogen atom, with subsequent deprotonation giving the key zwitterionic C(1)‐ammonium enolate intermediate. Reaction with a suitable electrophile (to exemplify, a Michael acceptor) forms the functionalised acyl ammonium intermediate. Finally, intramolecular catalyst turnover by the generated anion is used to release the product and regenerate the catalyst. This turnover approach has found widespread application for the synthesis of chiral heterocycles in formal cycloaddition processes with high stereocontrol.^[5b]^ A key requirement in this strategy is the need for a latent nucleophilic component to be incorporated within the electrophile that is used to turnover the catalyst via intramolecular cyclisation. Although powerful, this approach also represents a fundamental limitation in these processes where only cyclic products can be formed. When using electron deficient aryl ester precursors, an alternative catalyst turnover pathway can be accessed (Scheme 2 b).^[16]^ In this case, the aryloxide anion, released upon *N*‐acylation of the Lewis base, can react with the post‐reaction acyl ammonium ion. This approach presents a strategy for the formation of acyclic α‐functionalised products at the carboxylic acid oxidation level, significantly broadening the potential applicability of C(1)‐ammonium enolates in enantioselective catalysis.

**Figure 2:**
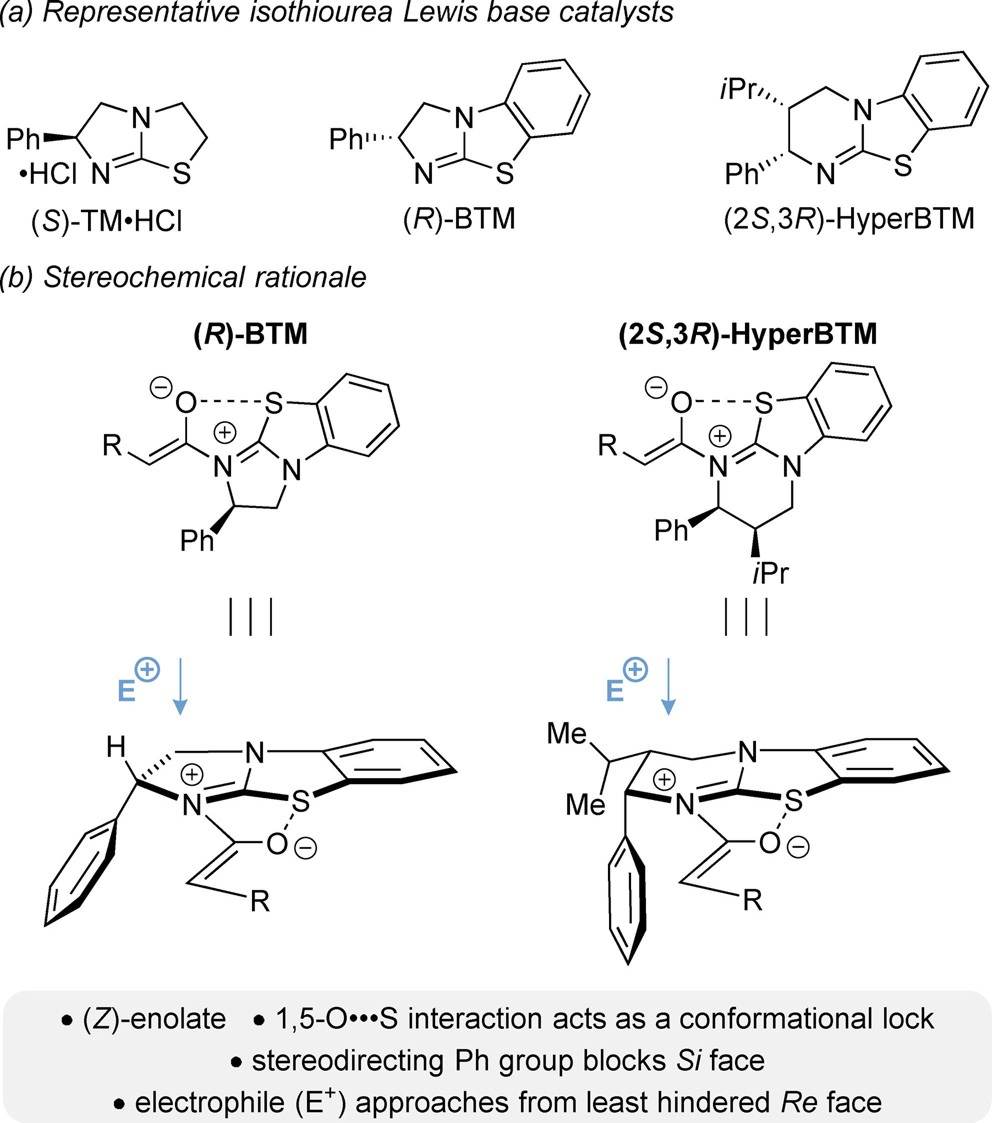
Isothiourea Lewis base organocatalysts.

Isothiourea Lewis base organocatalysts^[8]^ such as tetramisole (TM),^[17]^ benzotetramisole (BTM) and HyperBTM,[^18^, ^19^] have found widespread application in C(1)‐ammonium enolate catalysis, imparting high degrees of diastereo‐ and enantioselectivity (Figure 2 a). The enantiocontrol involving C(1)‐ ammonium enolates is governed by selective formation of the (*Z*)‐enolate and a 1,5‐O⋅⋅⋅S interaction (characterised as n_O_ to σ*_C‐S_) between the enolate oxygen anion and S atom of the catalyst,^[20]^ which restricts the conformational freedom of this intermediate (Figure 2 b). The stereodirecting phenyl group effectively blocks the *Si* face of the C(1)‐ammonium enolate, with preferential reaction with an electrophile occurring on the less hindered *Re* face.^[21]^

This review will survey selected recent advances in the generation and reactivity of C(1)‐ammonium enolates using isothiourea catalysis. For a comprehensive discussion on processes developed prior to 2014, readers are directed to a thorough previous review.^[5b]^ New concepts that allow access to C(1)‐ammonium enolates from different starting materials will be showcased. The application of both catalyst turnover methodologies highlighted in Scheme 2 in combination with new electrophilic partners for the synthesis of cyclic and acyclic α‐functionalised carboxylic acids, esters and amides will be featured. Key current concepts, including the development of more sustainable processes that do not require excess stoichiometric additives, will also be discussed. Advances in substrate scope will be highlighted, with particular focus on the α‐substituent of the enolate precursor, which has been traditionally limited to mono‐substitution with aryl, heteroaryl, alkenyl or heteroatom groups.

## 2 Catalyst Turnover via Lactonisation/Lactamisation

### 2.1 Intramolecular reactions

**Scheme 3:**
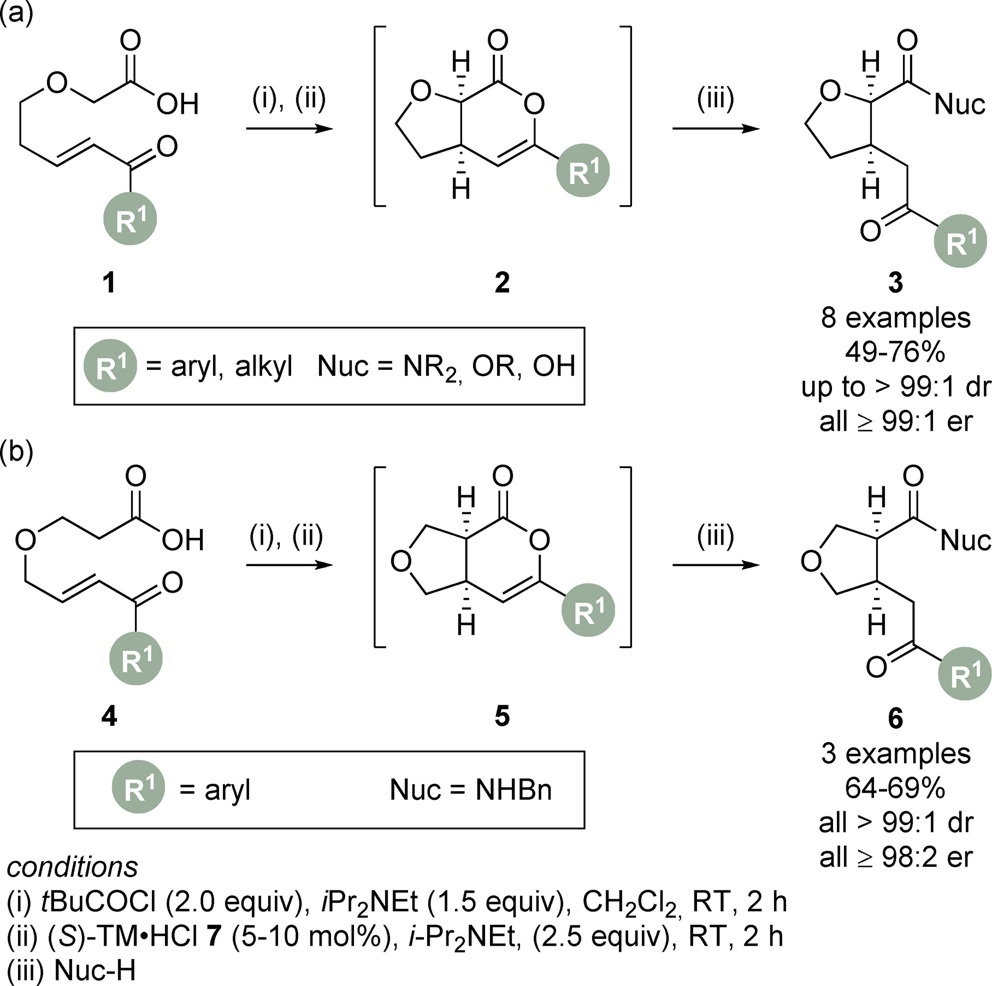
Michael addition/lactonisation for the synthesis of (a) *syn*‐2,3‐tetrahydrofurans and (b) *syn* 3,4‐tetrahydrofurans. (*S*)‐TM**⋅**HCl.

Smith and co‐workers reported the synthesis of *syn*‐2,3‐ and *syn*‐3,4‐tetrahydrofurans (THF)′s via a Michael‐addition/lactonisation cascade sequence (Scheme 3).^[22]^ Using 10 mol % (*S*)‐tetramisole **7** as the isothiourea catalyst, various 5‐oxo enone‐acids **1** were converted to the initial fused THF **2**, which proved unstable to chromatography. Subsequent addition of an external nucleophile (benzylamine, pyrrolidine, methanol, sodium hydroxide) enabled the corresponding ring‐opened *syn*‐2,3‐THF products **3** to be isolated in good yield with excellent stereocontrol (Scheme 3 a). Shifting the position of the oxygen tether by employing 4‐oxo enone‐acids **4** as starting materials, *syn*‐3,4‐THF products **6** could also be accessed under similar conditions (Scheme 3 b). As observed in previous work,^[12a]^ no acidifying substituent is required for reactivity in this case. It is proposed the intramolecular nature of this process means the C(1)‐ammonium can react efficiently despite only a low concentration of this intermediate being formed. By employing an alternative cinchona alkaloid Lewis base, the *anti*‐ diastereoisomer could be accessed in a stereodivergent protocol.

**Scheme 4:**
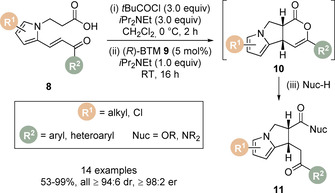
Michael addition/lactonisation for the synthesis of pyrrolizines.

This intramolecular protocol was extended to the catalytic enantioselective synthesis of pyrrolizines (Scheme 4).^[23]^ Using commercially available catalyst (*R*)‐BTM **9** (5 mol %), a range of pyrrole‐derived enone‐acids **8** were converted to dihydropyranones **10**. Following in situ ring‐opening with suitable amine and alcohol nucleophiles, the more stable pyrrolizine carboxylate derivatives **11** could be obtained in good to high yields (53–99 %) with excellent diastereo‐ and enantioselectivity (all ≥94:6 dr, ≥98:2 er). Notably, no competing Friedel‐Crafts acylation or β‐elimination of the pyrrole was observed in the presence of a mixed anhydride or acyl ammonium ion species. DFT calculations using the M06‐2X functional were applied to compute the energy profiles of reaction pathways of the (*Z*)‐C(1)‐ammonium enolate to form the *cis*‐ and *trans*‐isomers of dihydropyranones **10**. Significantly, formation of the observed *cis* isomer was computed to be both kinetically and thermodynamically favoured. Interestingly, an alternative pathway involving the (*E*)‐C(1)‐ammonium enolate was characterised but had a significantly higher barrier. Other observations included a calculated 1,5‐O⋅⋅⋅S distance to be shorter than the sum of the van der Waals radii found in all intermediates from the (*Z*)‐enolate onwards to catalyst dissociation, highlighting this non‐bonding interaction.

**Scheme 5:**
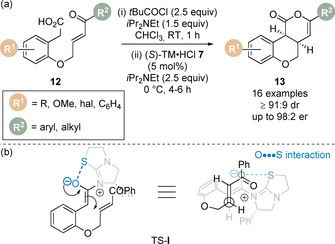
(a) 6‐*exo*‐trig Michael addition/lactonisation for the enantioselective synthesis of *cis*‐chromenones, and (b) stereochemical rationale.

The previous examples involve a common 5‐*exo*‐trig ring closure to give the corresponding heterocyclic product. Rarer is the related 6‐*exo*‐trig ring closure and there are only a limited number of examples of this transformation, all of which use cinchona alkaloid catalysts.[^11b^, ^11e^, ^11g^] The Michael addition/lactonisation methodology was therefore applied for the synthesis of chromenones via a 6‐*exo*‐trig cyclisation.^[24]^ Treatment of enone‐acids **12** with pivaloyl chloride and subsequent addition of tetramisole **7** (5 mol %) at 0 °C provided a suite of *cis*‐chromenones **13** in excellent yield, diastereomeric ratio (dr) and enantiomeric ratio (er) (Scheme 5 a). During detailed temporal studies it was observed that base‐catalysed epimerisation was in operation, leading to increased diastereomeric ratio and lower enantiomeric ratio upon extended reaction times. To circumvent this problem a post‐reaction acidic aqueous work‐up was incorporated to enable isolation of the products in high dr and er. The observed diastereo‐ and enantioselectivity of the Michael addition step can be rationalised by a simplistic model where the 1,5‐O⋅⋅⋅S interaction restricts the conformation of the (*Z*)‐enolate with Michael addition occurring *anti*‐ to the stereodirecting unit of the catalyst via pre‐transition state assembly TS‐**I** (Scheme 5 b).

### 2.2 Intermolecular reactions: Formal [4+2] cycloadditions

**Scheme 6:**
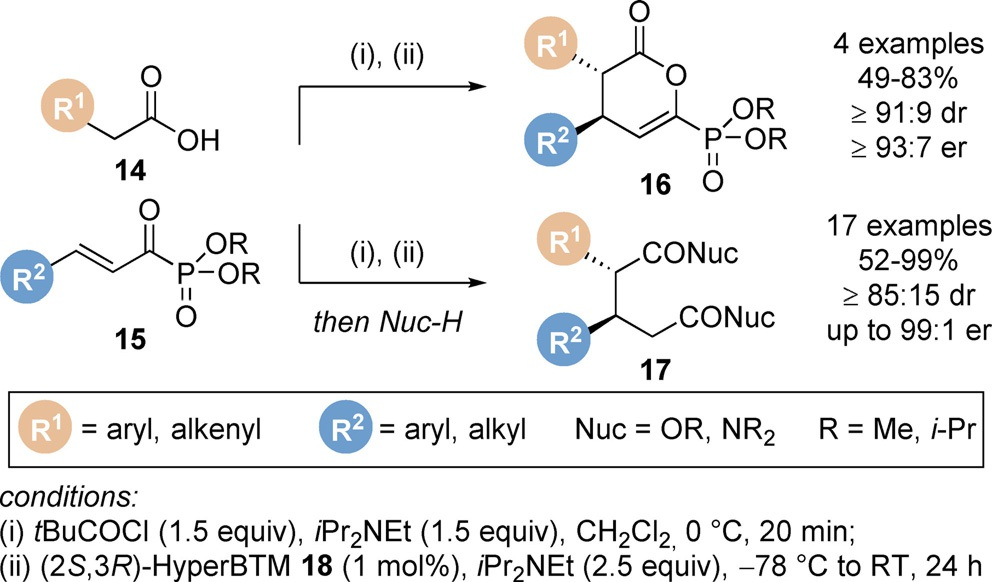
Michael addition/lactonisation using α‐keto‐β,γ‐unsaturated phosphonate Michael acceptors.

Effective intermolecular formal cycloadditions have been previously developed using highly electron‐deficient α‐keto‐β,γ‐unsaturated esters and trifluoromethyl enone Michael acceptors for enantioselective carbon‐carbon bond formation,[^12a^, ^12e^] whilst use of *N*‐aryl‐*N*‐aroyldiazene electrophiles have been investigated for carbon‐nitrogen bond formation.^[12c]^ This protocol was then extended to α‐keto‐β,γ‐unsaturated phosphonate electrophiles **15** as Michael acceptors in intermolecular Michael addition/lactonisation cascades (Scheme 6).^[25]^ Using aryl and alkenyl acetic acids as starting materials **14**, the derived C(1)‐ammonium enolates underwent formal [4+2] cycloaddition on reaction with the phosphonate Michael acceptors **15** to give stereodefined lactone products **16**. Treatment of the dihydropyranones **16** with a suitable nucleophile gave the corresponding 1,5‐diester or diamide **17**, exemplifying the ability of the phosphonate group to act as an ester surrogate. This protocol was later extended to use of trichloromethyl enone Michael acceptors as α,β‐unsaturated ester surrogates.^[26]^

**Scheme 7:**
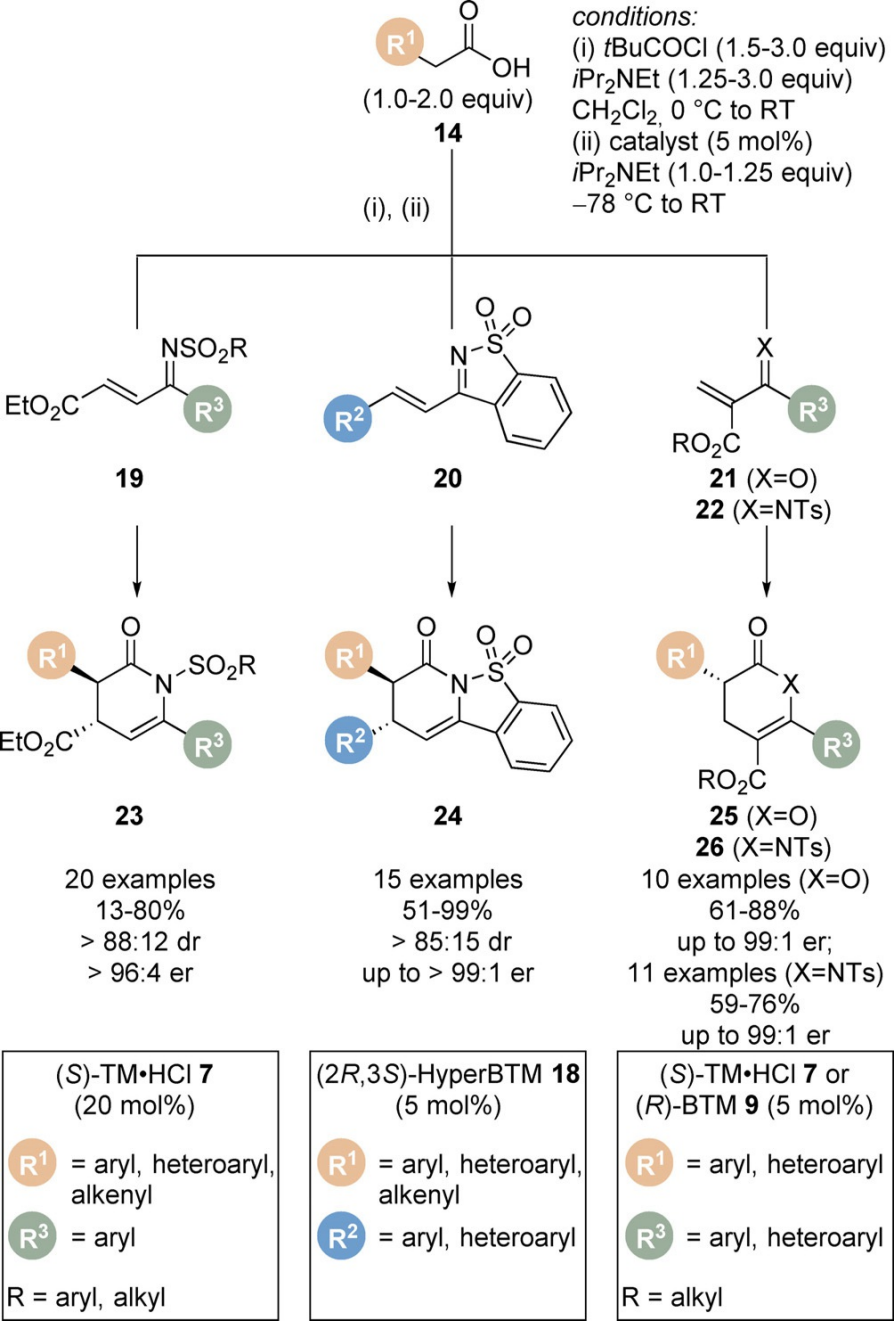
Michael addition/lactonisation or lactamisation using various Michael acceptors (when X=O: from homoanhydride).

Isothiourea‐catalysed Michael addition/lactamisation processes have also been disclosed (Scheme 7). Having been previously limited to chalcone‐based ketimine Michael acceptors,^[12b]^ the formal [4+2] cycloaddition with ketimines derived from α,β‐unsaturated γ‐ketoesters **19** to give a range of dihydropyridinones **23** was achieved in 2015.^[27]^ This process was extended to saccharin‐derived ketimines **20** as Michael acceptors in combination with aryl, heteroaryl and alkenyl acetic acids for the formation of polycyclic dihydropyridinones **24**.^[28]^ Typically, the formal [4+2] cycloaddition strategies yield the corresponding 3,4,6‐substituted dihydropyranones and dihydropyridinones. In 2016, the highly reactive β‐unsubstituted Michael acceptors **21** and **22** was used to construct the 3,5,6‐trisubstituted dihydropyranones and dihydropyridinones **25** and **26** containing a single stereogenic centre.^[29]^ Notably, a significant base‐mediated background reaction was observed when using 2‐aroylacrylate Michael acceptors **21** and carboxylic acids as starting materials. However, the use of homoanhydride enolate precursors enabled the preparation of corresponding products **25** with excellent enantioselectivity.

**Scheme 8:**
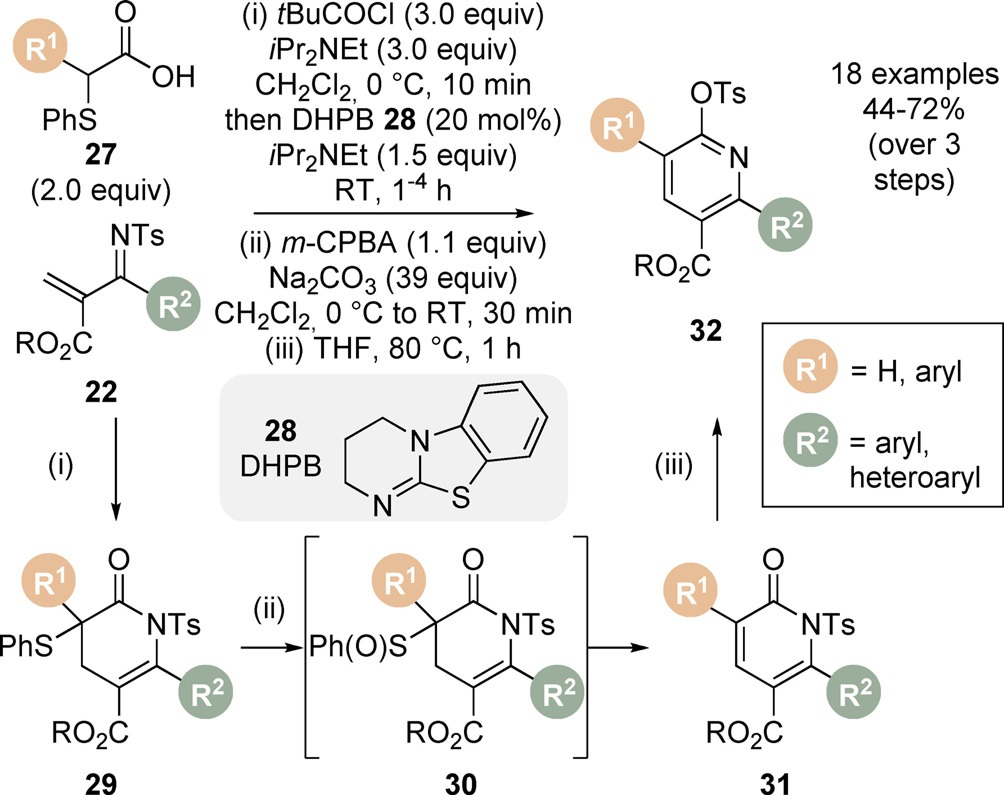
Isothiourea‐catalysed Michael addition/lactamisation, oxidation‐elimination sequence for the synthesis of 2,3,5,6‐tetrasubstituted pyridines. DHPB=dihydropyrimidobenzothiazole, *m*‐CPBA=*meta*‐chloroperoxybenzoic acid.

In 2014, the Michael addition/lactonisation methodology was incorporated into a sequence for the synthesis of di‐, tri‐ and tetrasubstituted pyridines (Scheme 8).^[30]^ Following the formal [4+2] cycloaddition between (phenylthio)acetic acids **27** and α,β‐unsaturated ketimines **22** catalysed by achiral isothiourea DHPB **28** to give dihydropyridinone products **29**, treatment with *m*‐CPBA yielded the desired sulfoxides **30**, which underwent elimination on warming to room temperature. The pyridones **31** were heated at 80 °C to provide the pyridine products **32**. However, attempts to carry out a one‐pot protocol were unsuccessful, yielding complex mixtures. Typically, α,α‐disubstituted acetic acid derivatives are unproductive starting materials for C(1)‐ammonium enolate generation using isothioureas. Notably in this case, α,α‐disubstituted (phenylthiol)acetic acids are tolerated, enabling substitution in the 5‐position of the pyridine. An advantage of this methodology is the 2‐tosyl functional handle in the product, which was utilised in a series of derivatisations (Heck, S_N_Ar).

**Scheme 9:**
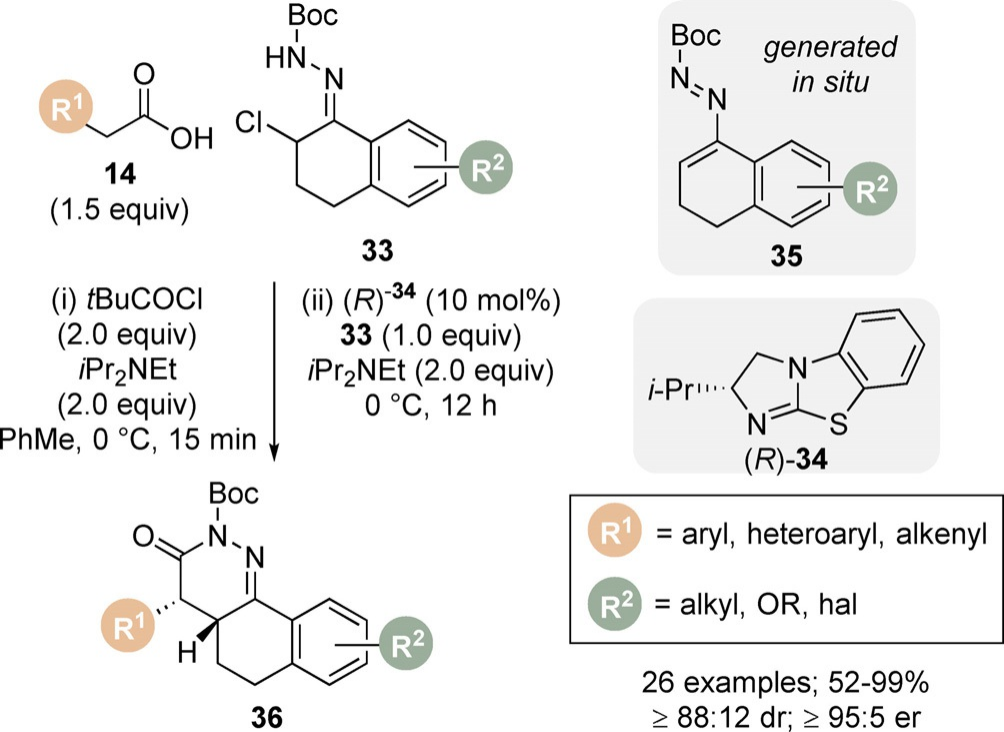
Isothiourea‐catalysed Michael addition/lactamisation of in situ generated azoalkenes. Boc=*tert*‐butlyoxycarbonyl.

Song and Zhang expanded the scope of the Michael addition/lactamisation protocol for the synthesis of enantioenriched dihydropyridazinone derivatives **36** (Scheme 9).^[31]^ It has been previously demonstrated that azoalkenes can be generated in situ under basic conditions from the corresponding α‐halogeno hydrazone.^[32]^ Inspired by Yao and co‐workers who reported the racemic synthesis of 4,5‐dihydropyridazin‐3(2*H*)‐ones using achiral isothiourea DHPB **28**,^[33]^ the authors employed this strategy in the formal [4+2] cycloaddition of carboxylic acids **14** and a range of cyclic α‐chloro *N*‐Boc hydrazones **33**, via azoalkene **35**, to furnish the 4,4a,5,6‐tetrahydrobenzo[h]cinnolin‐3(2*H*)‐one products **36** in high diastereo‐ and enantioselectivity using pivaloyl chloride as activating agent and (*R*)‐*i*Pr BTM catalyst **34**.

**Scheme 10:**
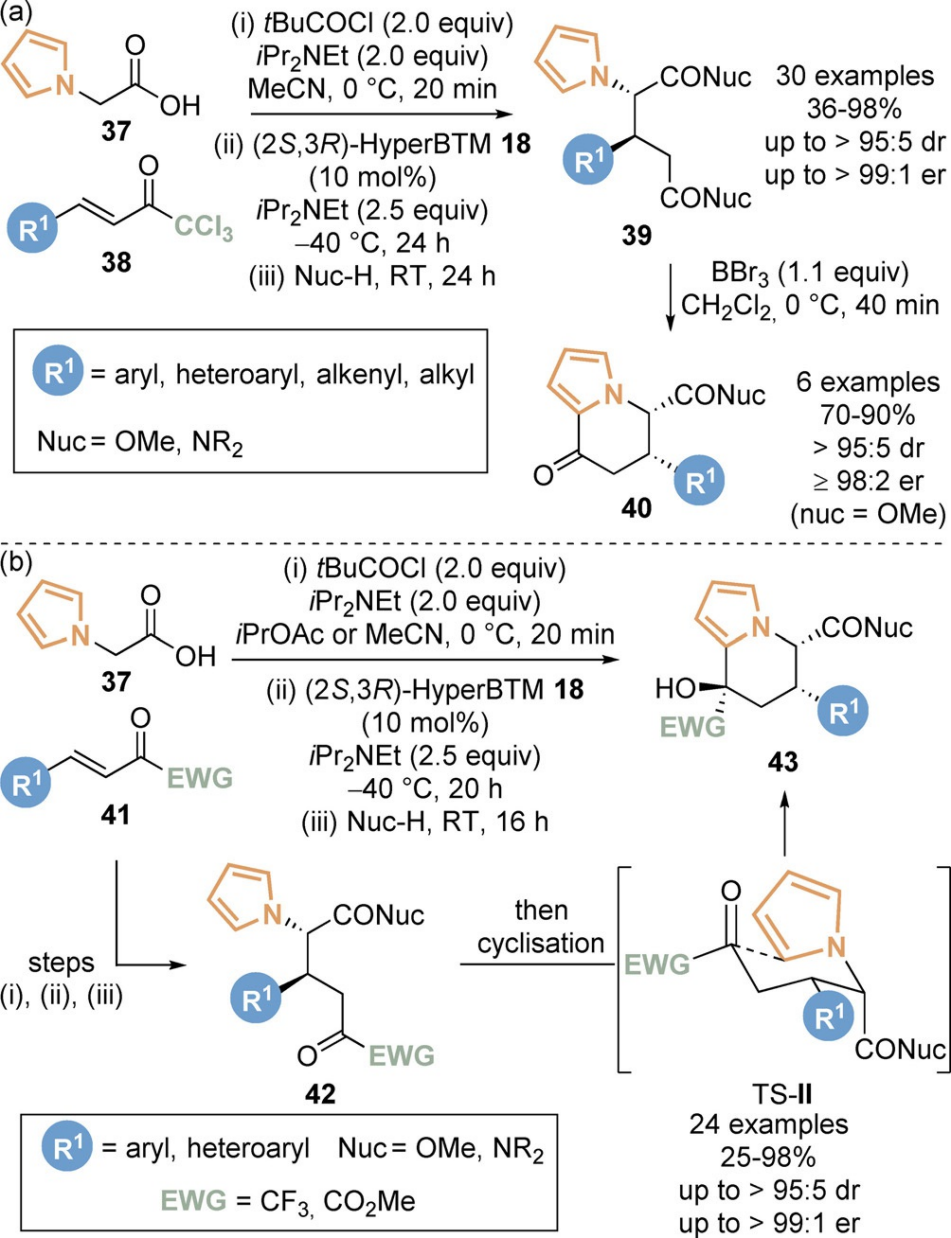
Isothiourea‐catalysed functionalisation of 2‐pyrrole acetic acid.

A key limitation in the reactivity of C(1)‐ammonium enolates derived from isothioureas is the typical requirement for an aryl, heteroaryl or alkenyl substituent in the C(2)‐position of the acetic acid nucleophile, with limited tolerance for heteroatom substituents. In 2018, an enantioselective functionalisation of 2‐pyrrole acetic acid **37** was developed (Scheme 10 a).^[34]^ Activation and Michael addition/lactonisation of pyrrole acetic acid **37** with a range of trichloromethyl enones **38**, followed by addition of an appropriate alcohol or amine nucleophile gave ring‐opened di‐ester and di‐amide products **39**. The nucleophilic pyrrole unit could be exploited in a series of Friedel–Crafts acylation derivatisations. Treatment of isolated diesters **39** with boron tribromide yielded functionalised dihydroindolizine **40** products whilst maintaining the diastereo‐ and enantiopurity.

Building upon this precedent, a one‐pot tandem sequential protocol was developed for the synthesis of enantioenriched tetrahydroindolizine derivatives **43** containing three stereogenic centres (Scheme 10 b).^[35]^ Michael addition/lactonisation reaction of 2‐pyrrole acetic acid **37** with Michael acceptors bearing an electron withdrawing group **41**, followed by ring opening by an appropriate amine or alcohol nucleophile, gave dicarbonyl intermediate **42**. It is proposed these can undergo spontaneous cyclisation of the pyrrole moiety onto the electrophilic ketone through chair‐like transition state TS‐**II**, yielding the tetrahydroindolizines **43** in high diastereo‐ and enantioselectivities. Key to the success of the protocol employing trifluoromethyl enone Michael acceptors was choice of reaction media; reactions carried out in acetonitrile and dichloromethane lead to a mixture of the desired product and by‐products from competing [2+2] formal cycloaddition and product isomerisation. Screening of 25 different solvents demonstrated that ethereal solvents were optimal for selectivity for the desired product, with diethyl ether, cyclopentylmethyl ether (CPME), ethyl acetate and isopropyl acetate giving best results.

**Scheme 11:**
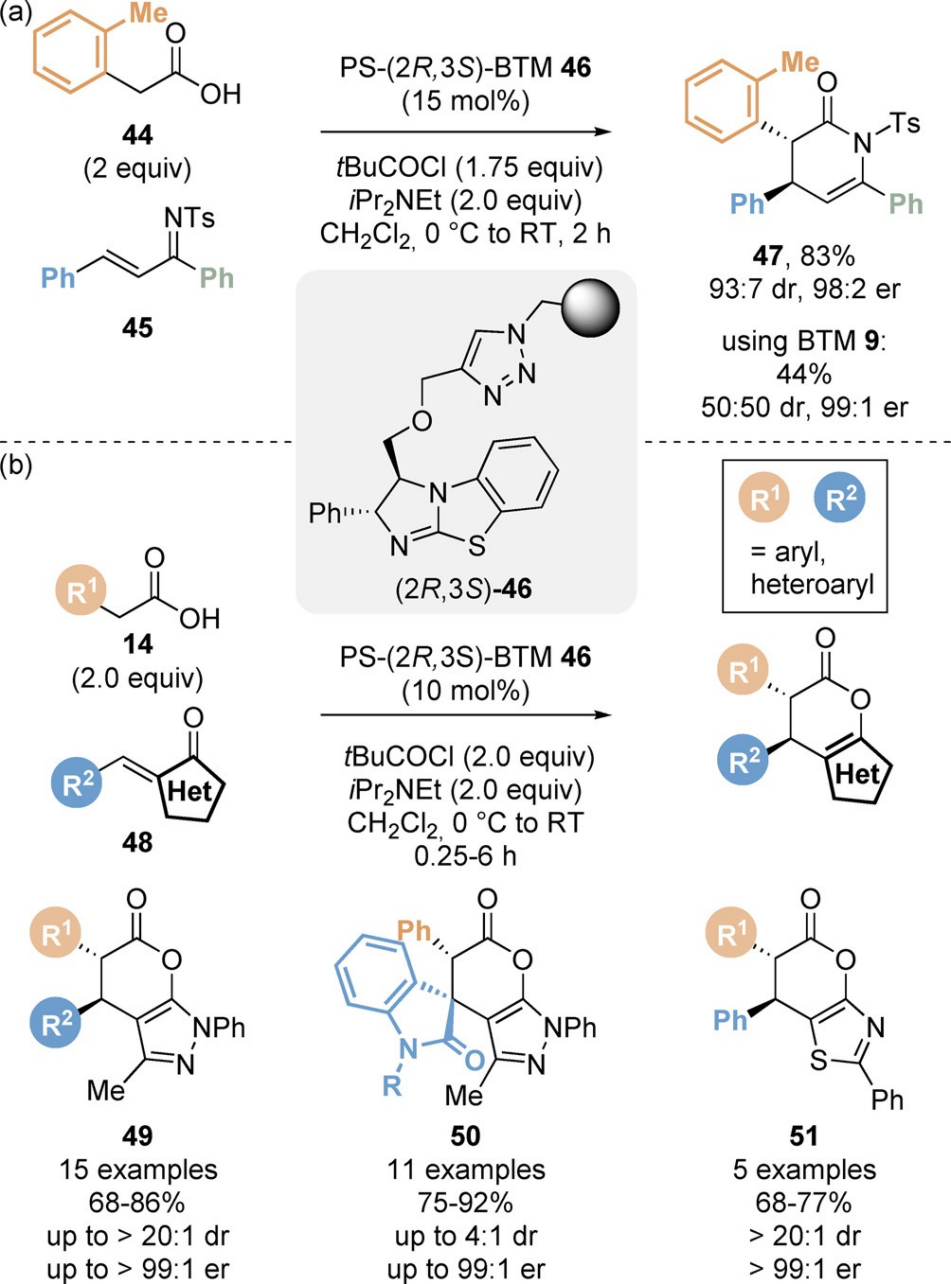
Enantioselective Michael addition/lactonisation catalysed by an immobilised isothiourea.

Pericàs and co‐workers demonstrated the first use of a polystyrene‐supported isothiourea **46** in a variety of Michael addition/cyclisation reactions (Scheme 11).^[36]^ First, the catalyst was tested in a benchmark Michael addition/lactamisation reaction of carboxylic acids with chalcone‐derived tosylimines (Scheme 11 a),^[36a]^ previously reported by Smith and co‐workers.^[12b]^ Notably, *ortho*‐substituted phenyl acetic acid derivatives such *ortho*‐tolyl **44** gave the corresponding dihydropyridone product **47** with enhanced diastereoselectivity (93:7 dr) compared to the corresponding reaction with BTM **9** (50:50 dr). It was proposed that substitution of the 3‐position of the catalyst to enable immobilisation destabilises the transition state leading to the minor *cis* diastereoisomer. During the optimisation of this process, it found that all the reagents could be combined from the start of the reaction and that premixing the carboxylic with pivaloyl chloride was not necessary, leading to a simplified protocol. Importantly, six consecutive cycles were performed with constant stereoselectivity and yield, exemplifying the recyclability of catalyst **46**. A continuous flow protocol was also developed through a sequential preactivation and reaction set‐up, which allowed 4.4 g of product to be obtained. This protocol was also applied in Michael addition/lactamisation reactions using saccharin‐derived sulfonyl imine electrophiles. In 2017, Pericàs and co‐workers extended the use of polystyrene‐supported isothiourea **46** to Michael addition/lactonisation reactions (Scheme 11 b).^[36b]^ Carboxylic acids **14** underwent smooth reaction with alkylidene pyrazolones, isatin derivatives and alkylidene thiazolones to give the corresponding polycyclic dihydropyranopyrazolones (**49**, **50**) and dihydropyranothiazolones **51**.

**Scheme 12:**
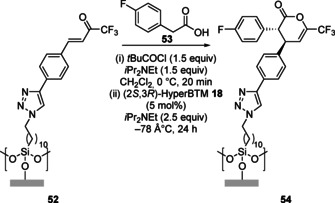
Direct Michael addition/lactonisation on a silicon oxide surface.

The Michael addition/lactonisation cascade methodology was employed by the groups of Smith and Hähner to enable the direct enantioselective functionalisation of a silicon oxide supported self‐assembled monolayer (Scheme 12).^[37]^ Reaction of surface‐bound trifluoromethyl enone **52** with 4‐fluorophenyl acetic acid **53** catalysed by HyperBTM **18** allowed the preparation of the modified surface **54**, with chiral AFM used to probe the enantioinduction on the surface.

**Scheme 13:**
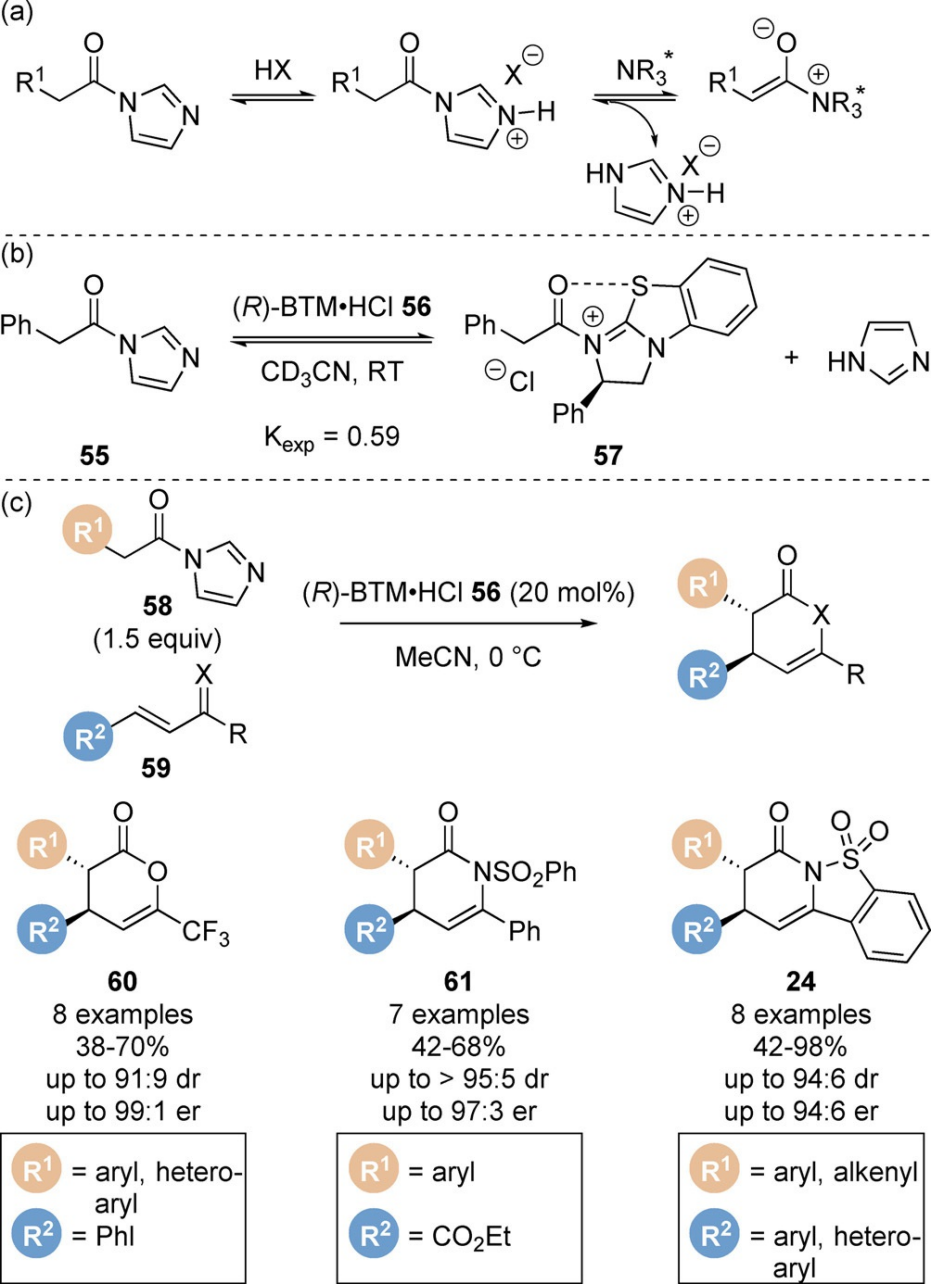
(a) The imidazolium effect for C(1)‐ammonium enolate generation, (b) initial equilibrium studies and (c) formal [4+2] cycloadditions.

**Scheme 14:**
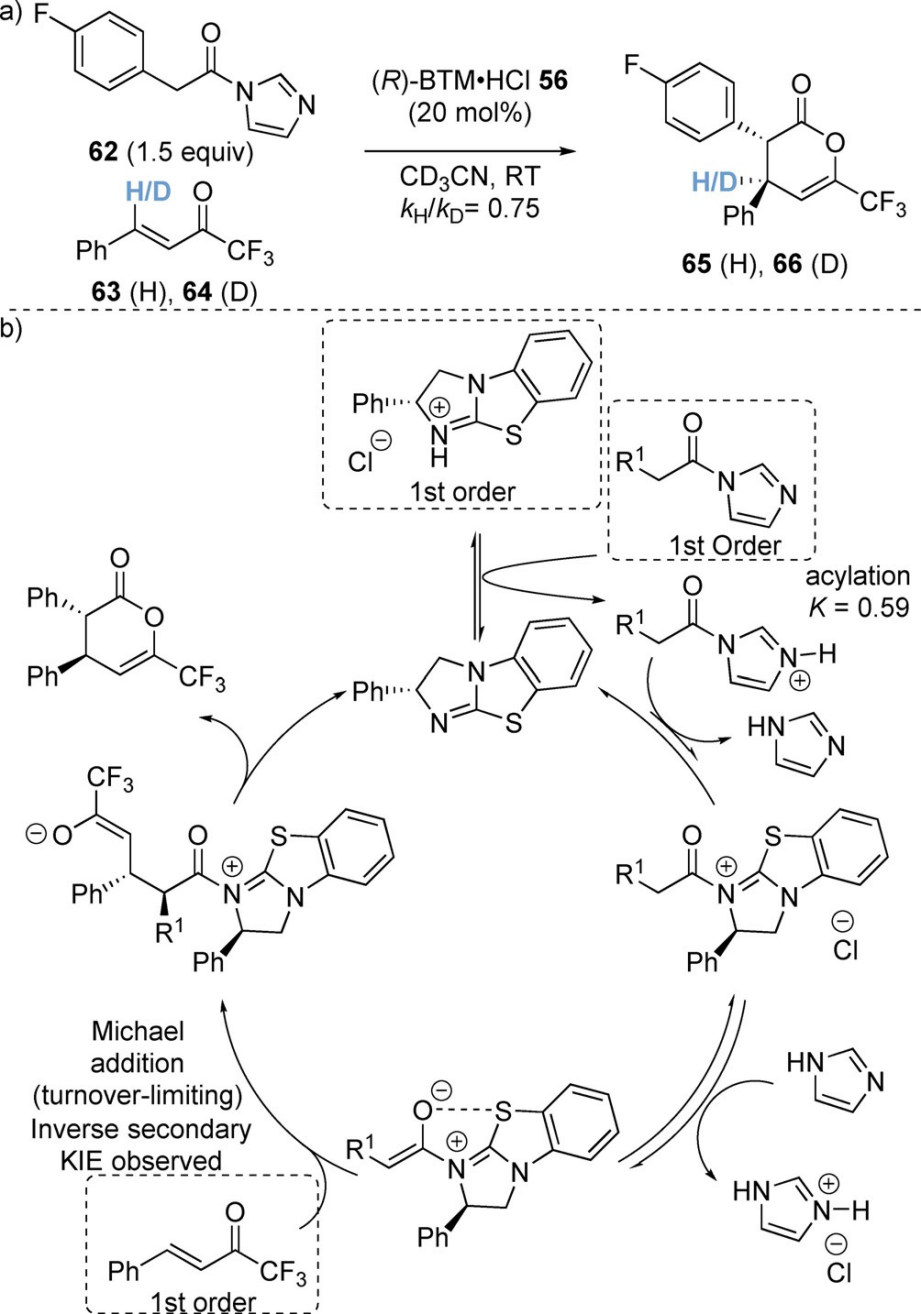
(a) Observed inverse secondary kinetic isotope effect (KIE) and (b) proposed mechanism using *N*‐acyl imidazoles.

**Scheme 15:**
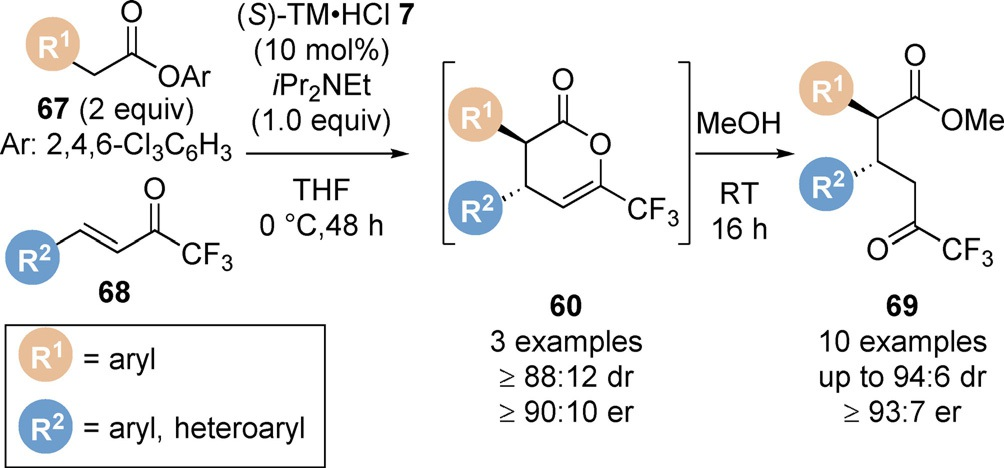
Michael addition/lactonisation using 2,4,6‐trichlorophenyl esters.

Whilst these strategies employing carboxylic acid precursors have been applied for the synthesis of a range of chiral heterocycles, one drawback is the use of stoichiometric (often multiple equivalents) of activating agent and auxiliary base to prepare a reactive acylating agent in situ. In addition, the by‐products generated from these processes (such as pivalic anhydride from pivaloyl chloride) can be difficult to separate from the desired products.^[12a]^ Considering this, Smith and co‐workers reported an alternative method of generating C(1)‐ammonium enolates from *N*‐acyl imidazoles under acidic conditions (Scheme 13 a).^[38]^ In situ protonation of the *N*‐acyl imidazole enables significantly enhanced catalyst acylation (utilising the recognised “imidazolium effect”)^[39]^ whilst the expected imidazole by‐product is non‐toxic and water soluble and can be readily removed from reaction mixtures. This was demonstrated in initial equilibrium studies between *N*‐acyl imidasole **55** and (*R*)‐BTM⋅HCl **56**, which led to rapid equilibration to *N*‐acyl ammonium ion pair **57** and imidazole with *K*_exp_=0.59 (Scheme 13 b). In contrast, no catalyst acylation was observed when acyl imidazole **55** and free base (*R*)‐BTM **9** were mixed. This was then exploited in formal [4+2] cycloadditions with a range of Michael acceptors **59** (trifluoromethyl enones, chalcone‐derived ketimines and saccharin‐derived ketimines). Comprehensive mechanistic investigations using ^19^F{^1^H} NMR reaction monitoring were carried out to provide further insight into the developed methodology. Kinetic analyses including reaction progress kinetic analysis (RPKA)^[40]^ and variable time normalisation analysis (VTNA)^[41]^ techniques were undertaken to determine the order of each component. The catalyst, *N*‐acyl imidazole and enone were found to be first order, whilst an inverse secondary kinetic isotope effect was observed (*k*_H_/*k*_D_=0.75), indicating the Michael addition step was turnover limiting (Scheme 14 a).^[42]^ Notably, same excess experiments found that the imidazole formed during the progress of the reaction inhibited the reaction rate. Based upon the information gained from the mechanistic studies, a catalytic cycle has been proposed (Scheme 14 b). In an extension of available bench stable enolate precursors, the use of aryl esters in the Michael addition/lactonisation approach has been reported (Scheme 15).^[43]^ Tetramisole **7** effectively catalyses the reaction of 2,4,6‐trichlorophenyl esters **67** with trifluoromethyl enones **68**.

**Scheme 16:**
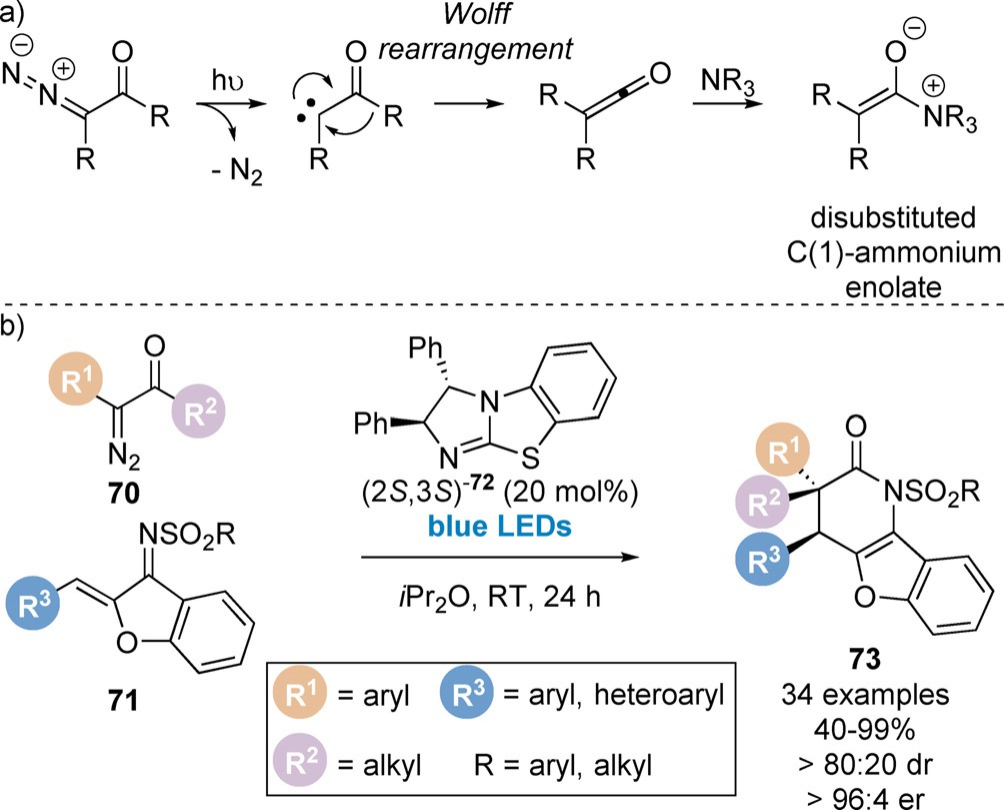
(a) Generation of C(1)‐ammonium enolates from α‐diazoketones and (b) application in Michael addition/lactamisation.

In 2019, Song and co‐workers, inspired by previous work by Lectka using cinchona alkaloid catalysts,^[44]^ developed an elegant method to generate isothiouronium enolates from α‐ diazoketones for the first time in combination with visible light photoactivation (Scheme 16).^[45]^ Excitation of the diazoketone initiates nitrogen extrusion to generate the α‐keto carbene intermediate which can undergo [1,2]‐migration to afford the ketene intermediate (Scheme 16 a). Significantly, this allows access to disubstituted ketenes, and therefore disubstituted C(1)‐ammonium enolates, which had previously been limited in this area of research using isothioureas. This strategy was applied in the enantioselective Michael addition/lactamisation of ketenes, generated from α‐diazoketones **70** and blue LED photoactivation, and aurone derived imine Michael acceptors **71**. The reaction was catalysed by a newly designed isothiourea catalyst **72**, enabling the construction of polycyclic benzofuran derivatives containing an all carbon quaternary stereogenic centre **73** (Scheme 16 b).

In 2020, the preparation of isourea **74** and isoselenourea **75** analogues of isothiourea **18** was reported and a comparative study used to probe the importance of 1,5‐Ch⋅⋅⋅Ch interactions in catalysis (Table 1).^[46]^ The ability of these catalysts was tested in a benchmark Michael addition/lactonisation process between acid **53** and Michael acceptor **63**. As postulated, isourea **74**, with weaker Ch⋅⋅⋅Ch interactions, performed poorest, giving product in modest yield with no enantioselectivity (entry 1). Catalysis using isothiourea **18** or isoselenourea **75** gave product in excellent yield, dr and er (entries 2,3). Significantly, when isoselenourea **75** was used the rate of product formation was significantly enhanced (*t*_1/2_=11 min) compared to isothiourea **18** (*t*_1/2_=122 min). These results demonstrate the increased stabilisation of catalytic intermediates with more favourable 1,5‐O⋅⋅⋅Ch interactions.

**Table 1:** Michael addition/lactonisation using isochalcogenourea catalysts.

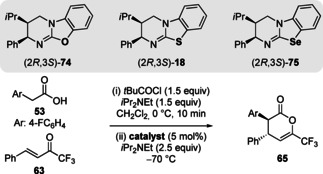					
Entry	Catalyst	Yield [%]	dr	er	[*t*_1/2_] [min]
1	O‐**74**	46	87:13	53:47	–
2	S‐**18**	80	84:16	95:5	122
3	Se‐**75**	95	84:16	98:2	11

### 2.3 Intermolecular reactions: Miscellaneous formal cycloadditions

**Scheme 17:**
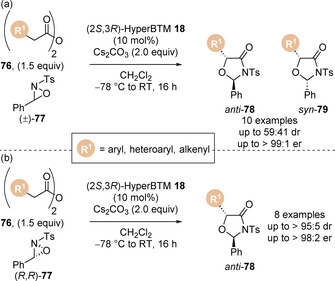
Isothiourea‐catalysed formal [3+2] cycloadditions with (a) racemic oxaziridines and (b) enantioenriched oxaziridines. Ts=tosyl.

Smith and co‐workers reported the formal [3+2] cycloaddition of homoanhydrides **76** with oxaziridine electrophiles **77** for the enantioselective preparation of oxazolidine‐4‐ones (Scheme 17 a).^[47]^ Using racemic oxaziridines **77** low diastereoselectivities were observed (up to 59:41 dr). Further investigations revealed the diastereoselectivity of the reaction decreased over time (80:20 to 60:40 dr), indicating a kinetic resolution process was occurring, with one enantiomer of the oxaziridine being consumed faster than its counterpart. Employing an enantioenriched oxaziridine **77** enabled the synthesis of a range of *anti*‐oxazolidin‐4‐ones **78** in high yield with excellent stereocontrol (Scheme 17 b). However, using the opposite enantiomer of the catalyst could only afford *syn*‐products in 80:20 dr due to mismatched effects.

**Scheme 18:**
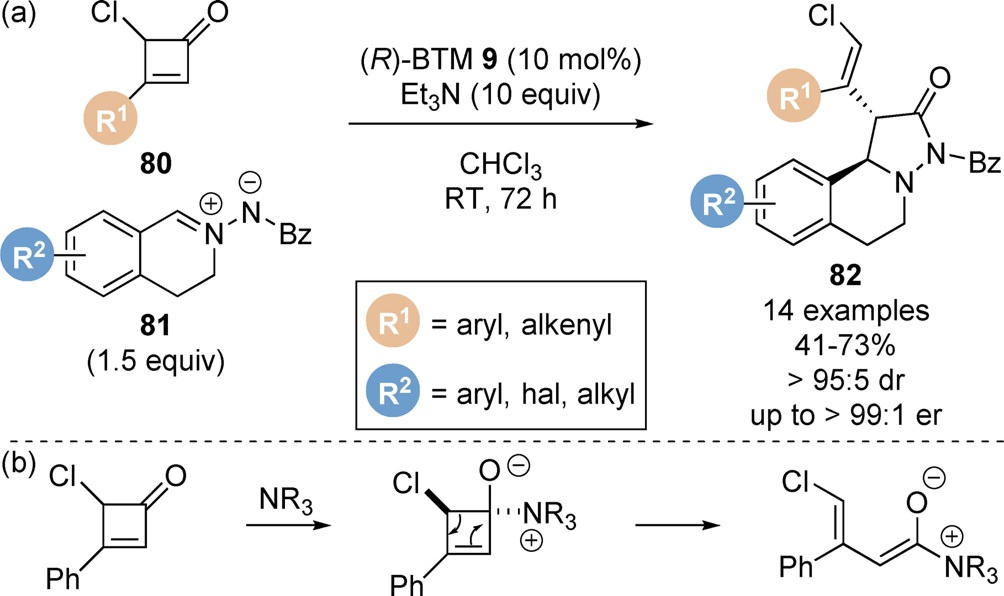
Isothiourea‐catalysed formal [3+2] cycloadditions of C(1)‐ammonium enolates generated from mono‐chloro‐substituted cyclobutenones. Bz=benzoyl.

**Scheme 19:**
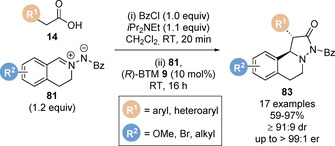
Isothiourea‐catalysed formal [3+2] cycloadditions of C(1)‐ammonium enolates generated from carboxylic acids.

In 2015, Chi and co‐workers reported the isothiourea‐catalysed formal [3+2] cycloaddition of C(1)‐ammonium enolates generated from mono‐chloro substituted cyclobutenones **80** and a range of azomethine imines **81** for the synthesis of highly functionalised enantioenriched pyrazolidinone derivatives **82** (Scheme 18 a).^[48]^ This is the first example of the generation of a C(1)‐ammonium enolate from a cyclobutanone starting material. It is postulated that the mechanism proceeds via 1,2‐addition of the isothiourea Lewis base to the mono‐chloro substituted cyclobutenone, breaking the conjugation of the starting material and generating anionic oxy‐intermediate (Scheme 18 b). Considering the (*Z*)‐configuration of the alkene and the absolute configuration at C(2) in product **82**, a *trans*‐relative stereochemistry between the chloro and isothiouronium substituents of this intermediate is likely but not defined in the original manuscript. This can undergo strain‐release driven carbon‐carbon bond cleavage through electrocyclic conrotatory 4πe ring‐opening to give the dienolate. The resultant dienolate reacts regioselectively through the α‐carbon, consistent with previous work in this area.^[12g]^ Notably, unsubstituted, methyl‐substituted and dichloro‐substituted cyclobutenones were unreactive in this protocol. Significantly, this process does not generate stoichiometric by‐products in the generation of the C(1)‐ammonium enolate, although 10 equivalents of auxiliary base are required. Studer and co‐workers described the related protocol of C(1)‐ammonium enolates generated from carboxylic acids **14** in the formal [3+2] cycloaddition with *C*,*N*‐cyclic azomethine imines **81** (Scheme 19).^[49]^ Additional DFT studies revealed a stepwise mechanism, with C−C bond formation being rate and stereochemical determining.

In 2017, Pericàs and co‐workers expanded the scope of the lactonisation/lactamisation approach to higher order enantioselective formal cycloadditions (Table 2).^[50]^ Employing immobilised polystyrene‐supported benzotetramisole catalyst **46**, phenyl carboxylic acid **84** underwent periselective formal [8+2] cycloaddition with azaheptafulvene electrophile **85** to give enantioenriched fused pyrrolidine‐cycloheptatriene **86** with excellent diastereo‐ and enantioselectivity (Table 2, entry 1). During the optimisation of this process, the use of a catalyst bearing two stereogenic centres was critical for high diastereoselectivity. When homogeneous isothiourea catalyst **9** bearing a single stereogenic centre was employed only moderate diastereoselectivity was observed (75:25 dr, Table 2, entry 2). Further studies revealed the importance of the auxiliary base in the reaction. Switching from DBU to tertiary amine bases such as Hünig's base or triethylamine led to a reduction in enantioselectivity (Table 2, entries 3 and 4). The developed conditions were then applied for the synthesis of a range of enantioenriched fused pyrrolidine‐cycloheptatriene products using a variety of carboxylic acids and azaheptafulvenes (19 examples, 37–85 %, >12:1 dr, up to 99:1 er). The authors also demonstrated the recyclability of the immobilised isothiourea catalyst, which was recovered by simple filtration. The catalyst could be used for four runs without loss in stereoselectivity and only marginal decrease in yield.

**Table 2:** Formal [8+2] cycloaddition catalysed by an immobilised isothiourea. DBU=1,8‐diazabicyclo[5.4.0]undec‐7‐ene.

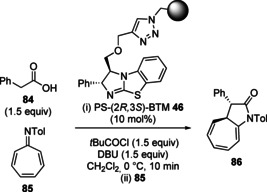				
Entry	Modification of conditions	Conv. [%]	dr	er
1	none	>95	96:4	96:4
2	(*R*)‐BTM **9** as catalyst	>95:5	75:25	96:4
3	*i*Pr_2_NEt as base	>95	96:4	94:6
4	Et_3_N as base	>95	96:4	85:15

**Scheme 20:**
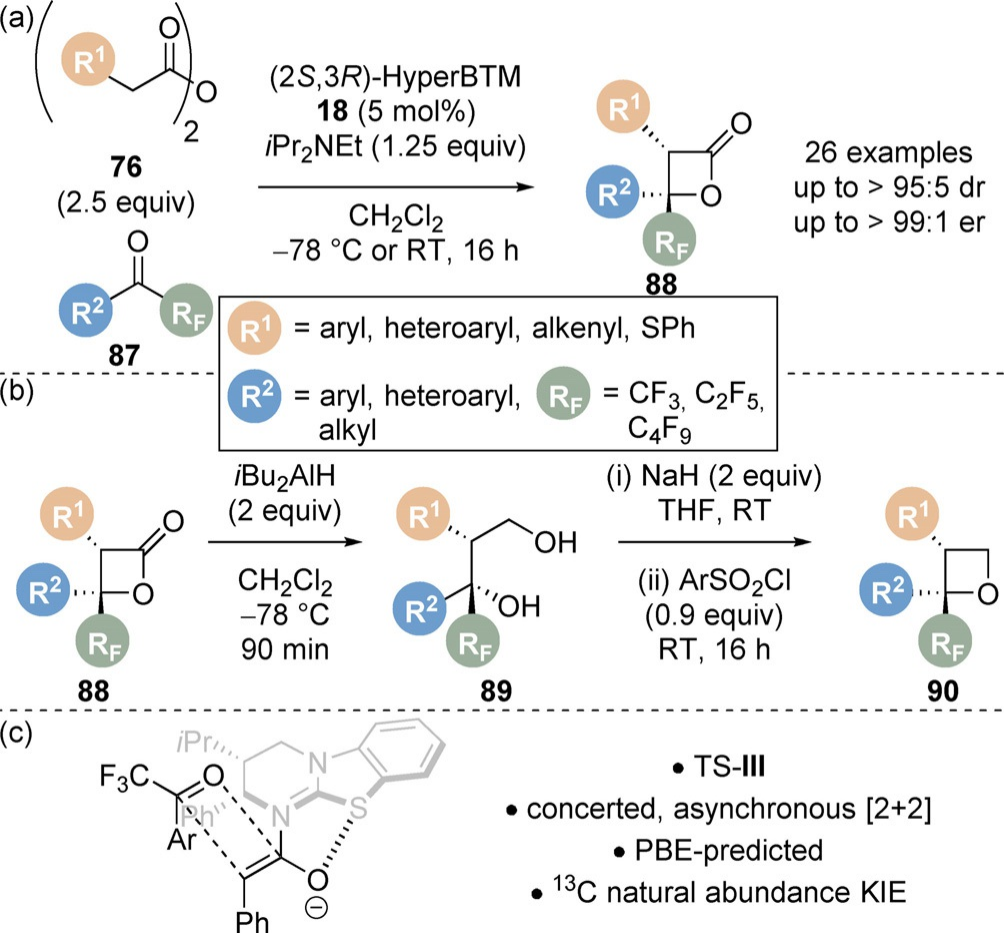
(a) Isothiourea‐catalysed synthesis of perfluoroalkyl‐substituted β‐lactones, (b) product derivatisation and (c) proposed transition state.

Formal [2+2] cycloaddition processes have also been disclosed.^[51]^ In 2019, the [2+2] cycloaddition of bench stable homoanhydrides **76** and perfluoroalkyl‐substituted ketones **87** for the preparation of enantioenriched perfluoroalkyl‐substituted β‐lactones **88** was reported (Scheme 20 a).^[51a]^ A wide reaction scope was demonstrated with aryl, heteroaryl, alkenyl and sulfide carboxylic acid derivatives tolerated along with aryl, heteroaryl and alkyl substituted perfluoroalkyl ketones. The synthetic utility of the products was demonstrated (Scheme 20 b) through a range of derivatisations including elaboration to the corresponding diol **89** (via reduction) and oxetane functionality **90** (through subsequent ring closure). Despite many reports of [2+2] cycloaddition of various C(1)‐ammonium enolate precursors with aldehyde and ketones, there were no mechanistic analyses of the reaction using tertiary amine catalysts reported. It was previously assumed to proceed via a stepwise aldol‐lactonisation pathway as opposed to a thermally forbidden concerted [2+2] cycloaddition. Computational studies using two alternate methods of density functional theory (DFT) predicted two different reaction pathways: stepwise aldol‐lactonisation (M06‐2X) and concerted asynchronous [2+2] (PBE). Experimental and computational natural abundance ^13^C kinetic isotope effect experiments revealed an excellent agreement for a rate‐limiting concerted asynchronous [2+2] cycloaddition via TS‐**III** (Scheme 20 c). Building on previous advances on the intermolecular formal [2+2] cycloaddition of carboxylic acids and *N*‐sulfonylimines,^[11h]^ Birman and co‐workers have reported the formal [2+2] cycloaddition of fluoroacetic acid and *N*‐sulfonylimines, allowing access to enantioenriched 3‐fluoro‐β‐lactams and α‐fluoro‐β‐amino acid derivatives.^[51b]^

### 2.4 Intermolecular reactions: Synergistic catalysis

**Scheme 21:**
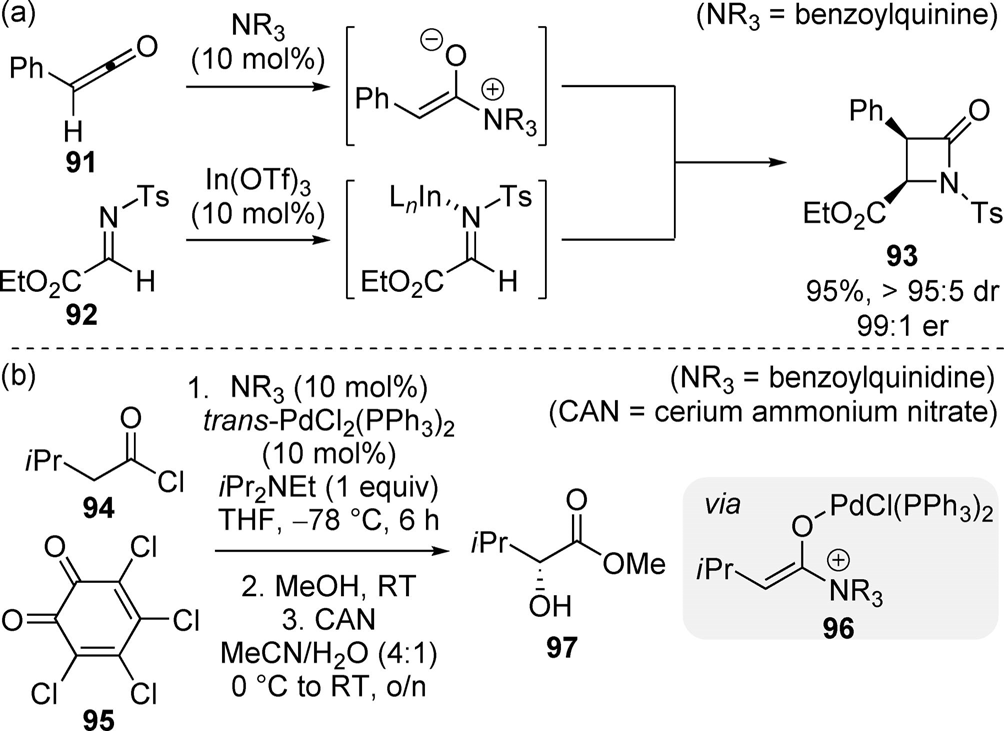
(a) Enantioselective synthesis of β‐lactams and (b) enantioselective α‐hydroxylation, enabled by dual tertiary amine/metal catalysis.

The unification of C(1)‐ammonium enolate intermediates with catalytically generated reaction partners in cooperative or synergistic processes has been targeted in the pursuit of novel reactivity modes. In 2002, in a pioneering contribution, Lectka and co‐workers introduced this concept in the diastereo‐ and enantioselective synthesis of β‐lactams **93** through dual Lewis base/Lewis acid catalysis (Scheme 21 a).^[52]^ A cinchona alkaloid‐based Lewis base catalyst was employed for the in situ generation of the C(1)‐ammonium enolate intermediate from a ketene precursor **91**, whilst an achiral Lewis acid catalyst (In(OTf)_3_) was proposed to increase the electrophilicity of the imine electrophile **92**. Subsequently, Lectka, and others, have demonstrated the utility of C(1)‐ammonium enolates combined with various metal Lewis acids.^[53]^ In another significant development, Leckta demonstrated the advantageous use of nickel, palladium and platinum Lewis acid catalysts in the reaction of C(1)‐ammonium enolates with *o*‐chloranil **95** for enantioselective α‐hydroxylation, with palladium giving optimal results (Scheme 21 b).^[54]^ In this case, rather than simple coordination to the electrophile to increase reactivity, it is proposed that the Lewis acid cocatalyst complexes to the C(1)‐ammonium enolate. This leads to stabilisation of this intermediate, increasing its concentration in the reaction mixture, therefore enhancing the rate of reaction.

**Scheme 22:**
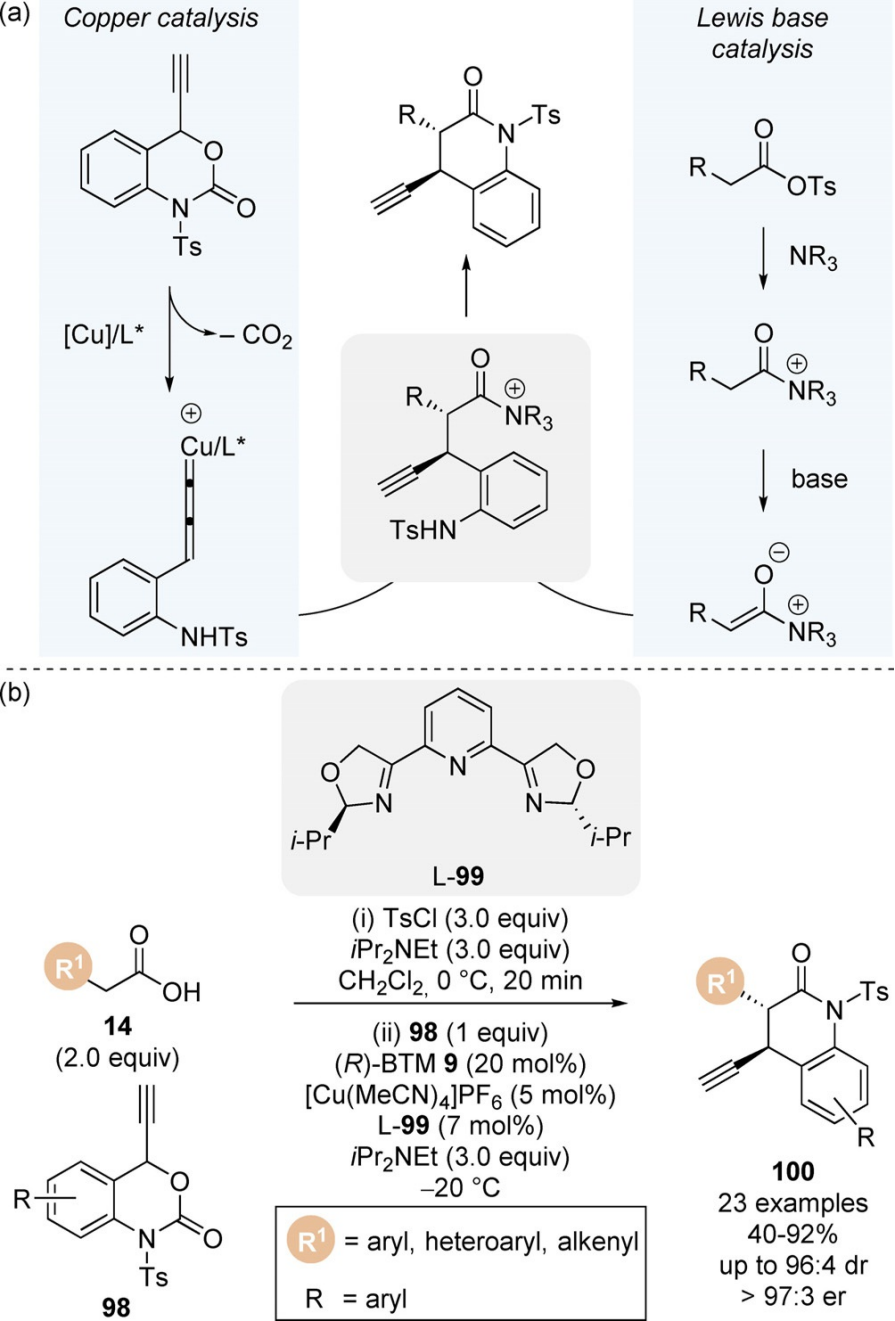
Dual copper/isothiourea‐catalysed decarboxylative formal [4+2] cycloaddition for the synthesis of 3,4‐dihydroquinolin‐2‐one derivatives.

Transition metals are capable of catalysing a broad range of transformations and have shown to be compatible with various organocatalysts in dual catalytic processes.^[55]^ Inspired by the work of Lectka, Snaddon and co‐workers reported the first synergistic isothiourea/transition metal catalysis process involving C(1)‐ammonium enolates intermediates in 2016 (discussed in section 3.2).^[56]^ Building on this precedent, Gong and co‐workers demonstrated the combination of an isothiourea Lewis base and copper catalytic cycles for the enantioselective α‐propargylation of carboxylic acids (Scheme 22 a).^[57]^ Uniting the transient C(1)‐ammonium enolate simultaneously with an electrophilic copper‐allenylidene complex generated through the known decarboxylation of propargylic ester derivatives enabled the formation of intermediate acyl ammonium,^[58]^ which could undergo lactamisation to form 3,4‐dihydroquinolin‐2‐ones. Using a chiral copper complex generated in situ from [Cu(MeCN)_4_]PF_6_] and pyridinyl bis(oxazoline) ligand **99**, the authors reported the formal [4+2] annulation of a range of carboxylic acids **14** and 4‐ethynyl dihydrobenzooxazinones **98** to give the 3,4‐dihydroquinolin‐2‐one products **100** with excellent stereoselectivities (Scheme 22 b). Lower diastereoselectivities were observed when achiral ligands were used, or when the opposite enantiomer of isothiourea was employed ((*S*)‐BTM **9**), indicating the matched chirality of each catalyst is crucial for stereocontrol. Wu and co‐workers have also developed a related α‐propargylation/lactamisation cascade using pivaloyl chloride to generate a reactive mixed anhydride in situ.^[59]^

**Scheme 23:**
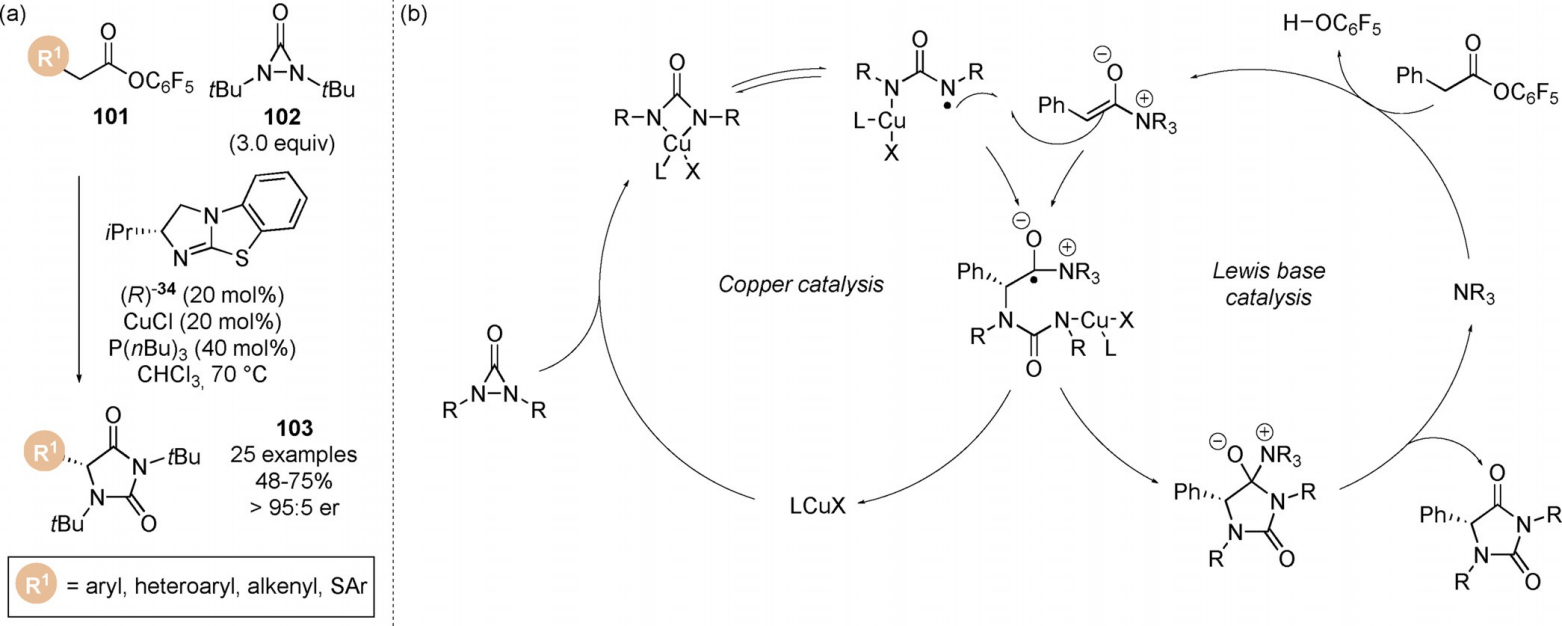
Cooperative copper/isothiourea‐catalysed α‐amination of esters using diaziridinone.

Inspired by work by Shi on the copper catalysed α‐amination of methyl esters,^[60]^ Gong and co‐workers rendered this transformation enantioselective through the development of a cooperative Lewis base and copper catalysis approach (Scheme 23 a).^[61]^ A range of pentafluorophenyl esters **101** underwent smooth α‐amination with *N*,*N*‐di‐*tert‐*butyldiaziridinone **102** to give hydantoin products **103** with excellent enantioselectivity when subjected to chiral (*R*)‐*i*Pr BTM **34** and Cu^I^ phosphine complex. Key to the success of this process was judicious choice of the electron deficient aryl ester. When pentafluorophenyl ester was used, significantly higher yields (and enantioselectivities) were observed compared to the corresponding *para*‐nitrophenyl ester, an observation first reported by Snaddon and co‐workers in 2016.^[56]^ A range of copper salts were found to work efficiently (CuCl, CuBr, CuI), however the phosphine ligand was required to enhance the catalytic activity of Cu^I^. The proposed mechanism involves N−N bond cleavage of *N*,*N*‐di‐*tert*‐butyldiaziridinone by the Cu^I^ catalyst to generate a four‐membered Cu^III^ species which is in equilibrium with a Cu^II^ radical species (Scheme 23 b). Simultaneously, the Lewis base could undergo acylation on reaction with the pentafluorophenyl ester, and subsequent deprotonation of the acyl ammonium ion would form the transient C(1)‐ammonium enolate intermediate. Union of the two catalytic cycles through single electron reaction of the C(1)‐ammonium enolate with Cu^II^‐intermediate furnishes the radical intermediate. Subsequent cyclisation regenerates both copper catalyst and chiral isothiourea and give the product hydantoin. To confirm this Cu^I^/Cu^II^ mechanistic hypothesis, electron paramagnetic resonance (EPR) spectroscopy studies indicated the formation of a nitrogen radical when *N*,*N*‐di‐*tert*‐butyldiaziridinone **102** was treated with CuCl‐P(*n*Bu)_3_, which was also observed during monitoring of the reaction mixture. Due to the complex equilibria of copper catalysis, an alternative Cu^I^/Cu^III^ pathway via reaction of an acyl ammonium ion with Cu^III^ species followed by reductive elimination cannot be completely ruled out.

**Scheme 24:**
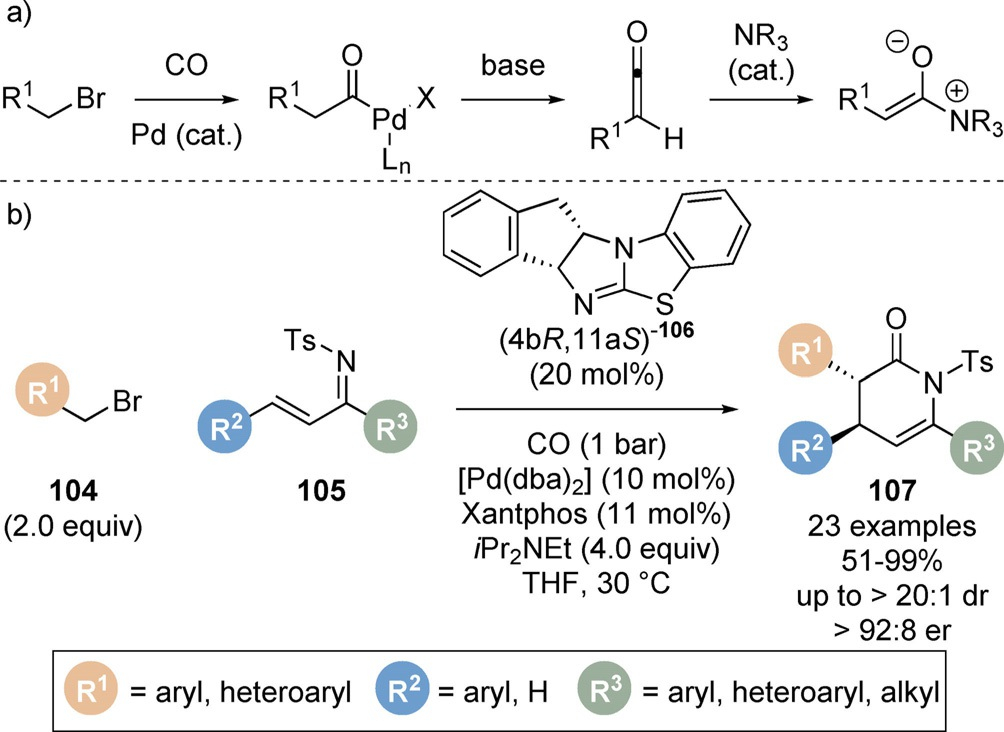
(a) Generation of C(1)‐ammonium enolates via palladium‐catalysed carbonylation and (b) application in a combined carbonylation/cycloaddition cascade.

Gong and co‐workers have also reported the seminal catalytic generation of C(1)‐ammonium enolates from the simple feedstock chemicals alkyl halides and carbon monoxide (CO).^[62]^ The authors hypothesised that oxidative addition of the palladium catalyst into the C−X bond of an alkyl halide, followed by CO insertion would give an acyl palladium intermediate (Scheme 24 a). Under basic reaction conditions a ketene intermediate could be accessed and intercepted by a Lewis base to generate the C(1)‐ammonium enolate. The authors demonstrated this protocol in a one‐pot palladium catalysed carbonylation‐Michael addition/lactonisation cascade for the formation of a range of dihydropyridones **107** (Scheme 24 b). This was also extended for the synthesis of β‐lactams on reaction with *N*‐tosyl imine electrophiles, enabling the rapid formation of molecular complexity from feedstock chemicals with high efficiency. It was found that lower pressures of CO were beneficial for the reaction. Whilst higher CO pressures favour CO insertion, high CO concentrations may limit oxidative addition and ketene formation by coordination to palladium. Catalyst **106** was identified as the optimal Lewis base catalyst, giving the products in higher diastereo‐ and enantioselectivity.

Although powerful in concept, catalyst turnover by lactonisation/lactamisation has limitations in terms of atom economy. When using carboxylic acid starting materials, pretreatment with stoichiometric amounts of activating agent and base is required to generate a reactive mixed anhydride in situ prior to catalyst acylation. In addition, an electrophile with a latent nucleophilic site is necessary for catalyst turnover via intramolecular cyclisation, limiting these methodologies to the formation of cyclic products. To overcome these shortfalls, recent work has focused on a novel catalyst turnover method using aryloxides that is exemplified in section 3.

## 3 Catalyst Turnover via Aryloxide

**Scheme 25:**
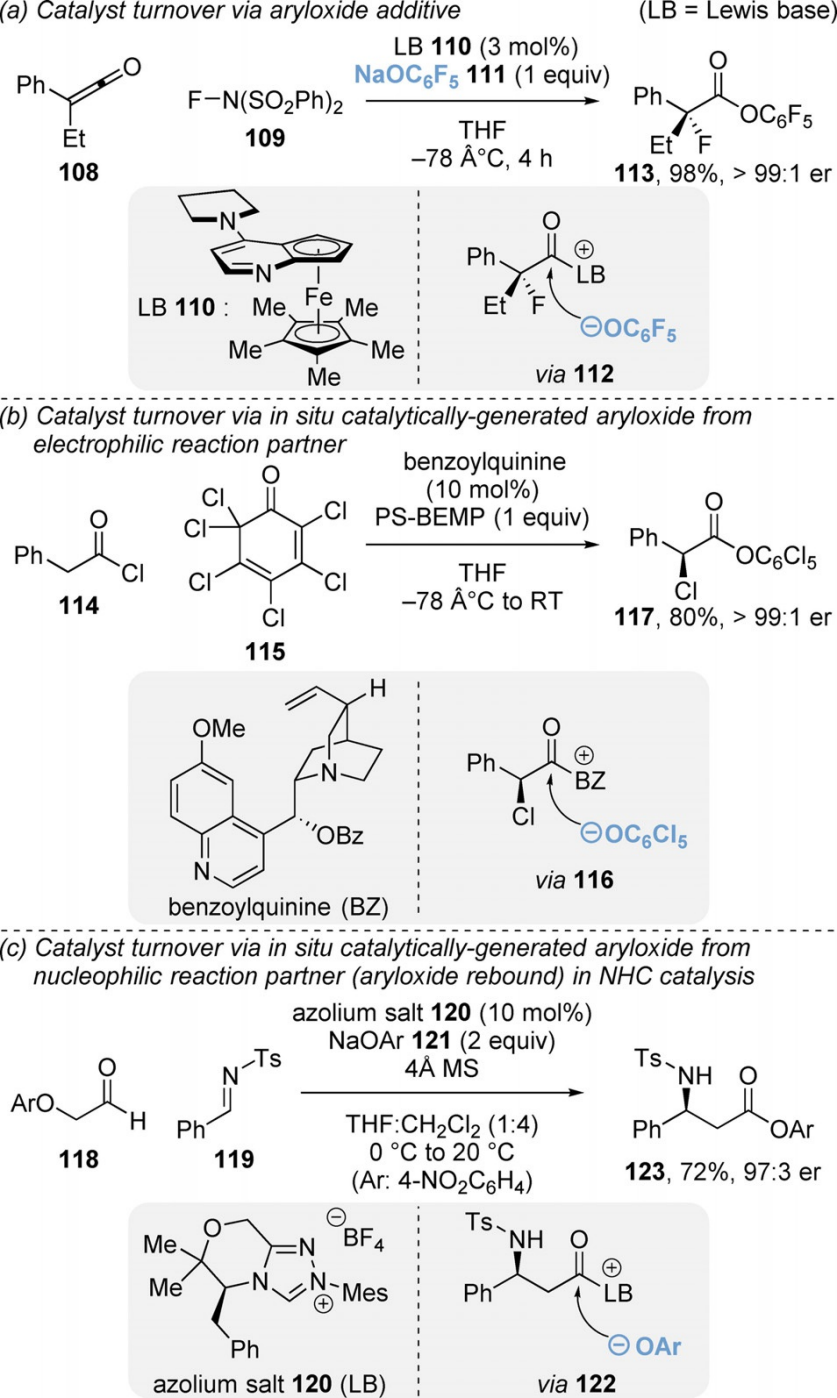
Catalyst turnover via (a) aryloxide additive, (b) aryloxide generated from electrophilic reaction partner, and (c) aryloxide generated from nucleophilic reaction partner. Mes=mesityl.

Catalyst turnover via aryloxide relies on the intermolecular addition of an aryloxide nucleophile to the post‐reaction acyl ammonium ion to complete the catalytic cycle (Section 1, Scheme 2 right). One method to achieve this is the inclusion of a stoichiometric aryloxide as an additive. In 2014, Fu and co‐workers utilised sodium pentafluorophenolate **111** as a catalyst turnover agent for the enantioselective fluorination of ketenes **108** using planar chiral DMAP catalyst **110** via a C(1)‐ammonium enolate intermediate (Scheme 25 a).^[63]^ However, this approach requires the aryloxide to be compatible with other reagents, or, as in this case, the dropwise addition of reagents to avoid side reactions.

Alternatively, the aryloxide can be catalytically generated in situ from a reaction partner. This was first established in conjunction with C(1)‐ammonium enolate intermediates in a series of elegant manuscripts by Leckta and co‐workers for enantioselective halogenation.[^10a^, ^64^] Reaction of the C(1)‐ammonium enolate (generated from ketenes) with a polyhalogenated quinone electrophile **115** gave the acyl ammonium/aryloxide ion pair, with the aryloxide used for catalyst turnover (Scheme 25 b). Scheidt and co‐workers have developed a related “aryloxide rebound” concept in an NHC‐catalysed formal Mannich process.^[65]^ Opposite to Lectka's strategy, in this case the aryloxide is generated from the nucleophilic reaction partner. Using α‐aryloxyaldehydes **118** as azolium enolate precursors, the aryloxide generated in situ can react with the post‐reaction acyl azolium ion to affect catalyst turnover (Scheme 25 c). It was proposed this approach could be translated to reactions of C(1)‐ammonium enolates through use of aryl ester enolate precursors.

### 3.1 Rearrangements of ammonium ylides

**Scheme 26:**
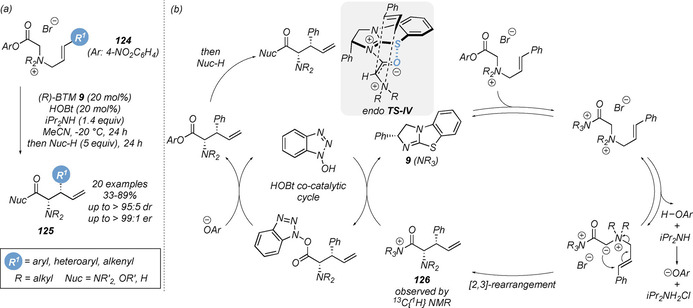
(a) Isothiourea‐catalysed asymmetric [2,3]‐sigmatropic rearrangement of allylic ammonium ylides and (b) the proposed mechanism.

In 2014, Smith and co‐workers reported the isothiourea‐promoted intramolecular [2,3]‐sigmatropic rearrangement of allylic quaternary ammonium salts **124** to give stereodefined *syn* α‐amino acid derivatives **125** bearing two contiguous stereogenic centres in excellent yield and stereoselectivity (Scheme 26 a).^[66]^ Key to optimal diastereo‐ and enantioselectivity was the addition of hydroxybenzotriazole (HOBt) as a co‐catalyst (61 % yield, 92:8 dr and 98:2 er without HOBt, vs. 76 % yield >95:5 dr and >99:1 er with HOBt). Various nucleophiles such as amines, alcohols and hydrides could be employed to give the corresponding amide, ester or alcohol products. To circumvent the need for salt isolation, a one‐pot allylic alkylation/[2,3]‐rearrangement protocol was also developed, although the products were isolated in diminished yield and enantioselectivity. Subsequent experimental and computational studies were also carried out to probe the reaction mechanism in detail (Scheme 26 b).^[67]^ Through extensive ^13^C and ^15^N isotopic‐labelling experiments and ^13^C{^1^H} NMR, post‐rearrangement intermediate **126** was identified as a catalyst resting state in the absence of HOBt. The addition of HOBt was found to shift the catalyst speciation toward the free catalyst, leading to increased catalyst concentration in the reaction mixture. The beneficial effect of HOBt was therefore proposed to originate from a higher concentration of free catalyst, enabling the enantioselective pathway to better outcompete the racemic background reaction. The reaction mechanism is proposed to proceed by direct and reversible *N*‐acylation of the catalyst by the *para*‐nitrophenyl ester ammonium salt to give the acyl ammonium ion intermediate and release *para*‐nitrophenoxide. Reversible deprotonation yields the ammonium ylide, which undergoes irreversible [2,3]‐sigmatropic rearrangement to give post‐rearrangement isothiouronium **126**. The rearrangement is both stereo‐ and product determining. Catalyst turnover is achieved by addition of HOBt to give the HOBt‐ester in a secondary co‐catalytic cycle, with rebound of the *para*‐nitrophenoxide giving the ester product that is subsequently converted to the corresponding amide by addition of an amine nucleophile. The observed stereoselectivity can be rationalised by an *endo* pretransition state assembly TS‐**IV** where the ammonium ylide exhibits the expected stabilising n_O_ to σ*_C‐S_ interaction alongside an additional π‐cation interaction between the allylic C(3)‐aryl substituent and the acyl isothiouronium ion.^[68]^ The rearrangement occurs from the opposite face to the stereodirecting phenyl unit of the catalyst.

**Scheme 27:**
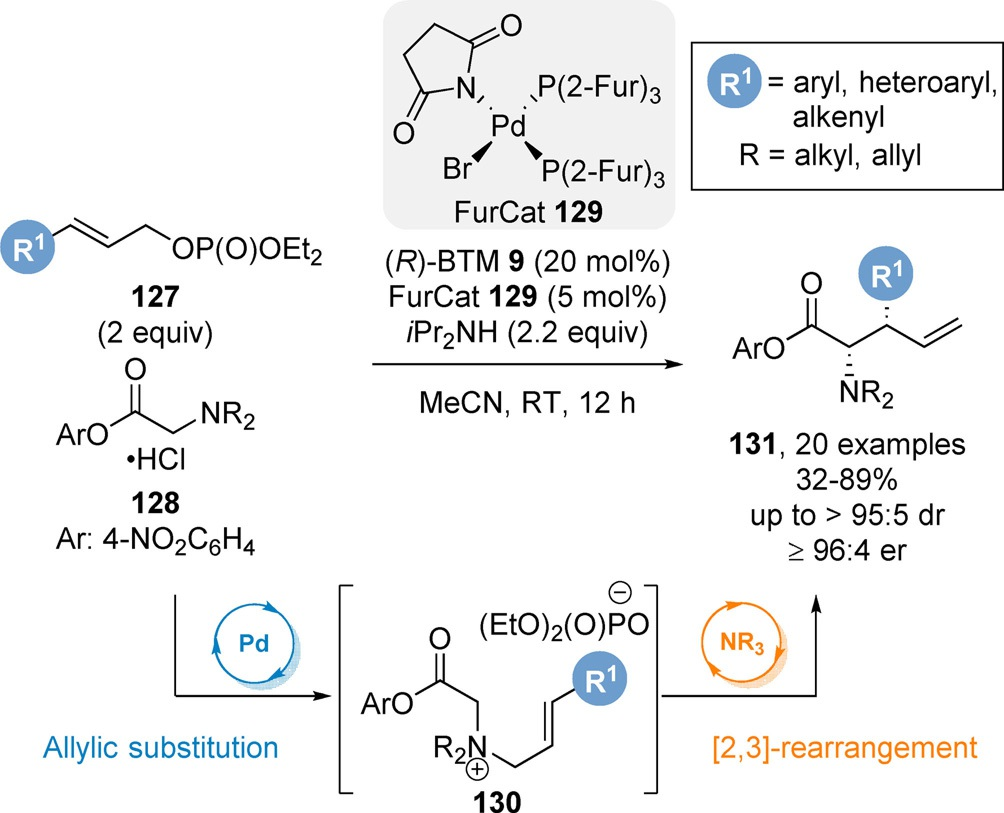
Synthesis of *syn* α‐amino acid derivatives via tandem palladium/isothiourea relay catalysis.

To overcome the challenge of isolating allylic quaternary ammonium salts, a tandem palladium/isothiourea relay‐ catalysed protocol was developed (Scheme 27).^[69]^ Combination of a palladium‐catalysed allylic amination of allylic phosphates **127** with glycine aryl ester derivatives **128** followed by a [2,3]‐rearrangement of intermediate **130** allowed direct access to a range of α‐amino acid derivatives **131**. Electron‐withdrawing phosphine ligands on palladium gave best reactivity, with defined succinimide‐based Pd complex (Furcat) **129** previously developed by Fairlamb proving the optimal palladium catalyst.^[70]^ Notably, the yields and stereoselectivities of this dual‐catalytic process are higher than that from the isolated allylic ammonium salts **124**. Unsymmetrical *N*,*N*‐dialkylglycine esters were also tolerated in this protocol, rearranging with high stereoselectivity, though these substrates are proposed to proceed via an intermediate acyl ammonium ylide containing a stereogenic nitrogen centre.

**Scheme 28:**
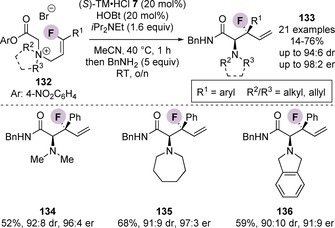
Enantioselective synthesis of β‐fluoro‐β‐aryl‐α‐aminopenten‐amides via [2,3]‐rearrangement of ammonium salts bearing a (*Z*)‐3‐fluoro‐3‐arylprop‐2‐ene group. Bn=benzyl.

**Scheme 29:**
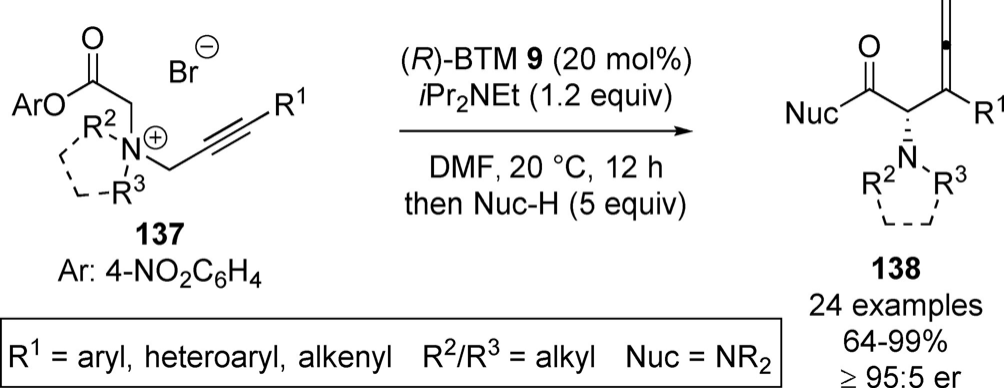
Asymmetric [2,3]‐rearrangement of propargylic ammonium salts.

This methodology was extended to the enantioselective [2,3]‐rearrangement of (*Z*)‐3‐fluoro‐3‐arylprop‐2‐ene containing quaternary ammonium salts **132**, enabling the synthesis of a range of β‐fluoro‐β‐aryl‐α‐aminopentenamides **133** containing a stereogenic tertiary fluorocarbon centre in high diastereo‐ and enantioselectivity (Scheme 28).^[71]^ Song and co‐workers later reported the first catalytic enantioselective [2,3]‐rearrangement of propargylic ammonium salt substrates **137**, leading to the enantioselective formation of allenyl α‐amino amides **138** in good yields with excellent enantioselectivity (Scheme 29).^[72]^ The process was tolerant of a range of aryl substituted propargyl units. However, alkyl and terminal alkyne containing substrates did not give the corresponding rearrangement product. A simplified one‐pot protocol was also developed, although this resulted in significantly diminished yields and lower enantioselectivity (60 % and 96:4 er, vs. 99 % and 97:3 er).

### 3.2 α‐Functionalisation of esters: Synergistic catalysis

**Scheme 30:**
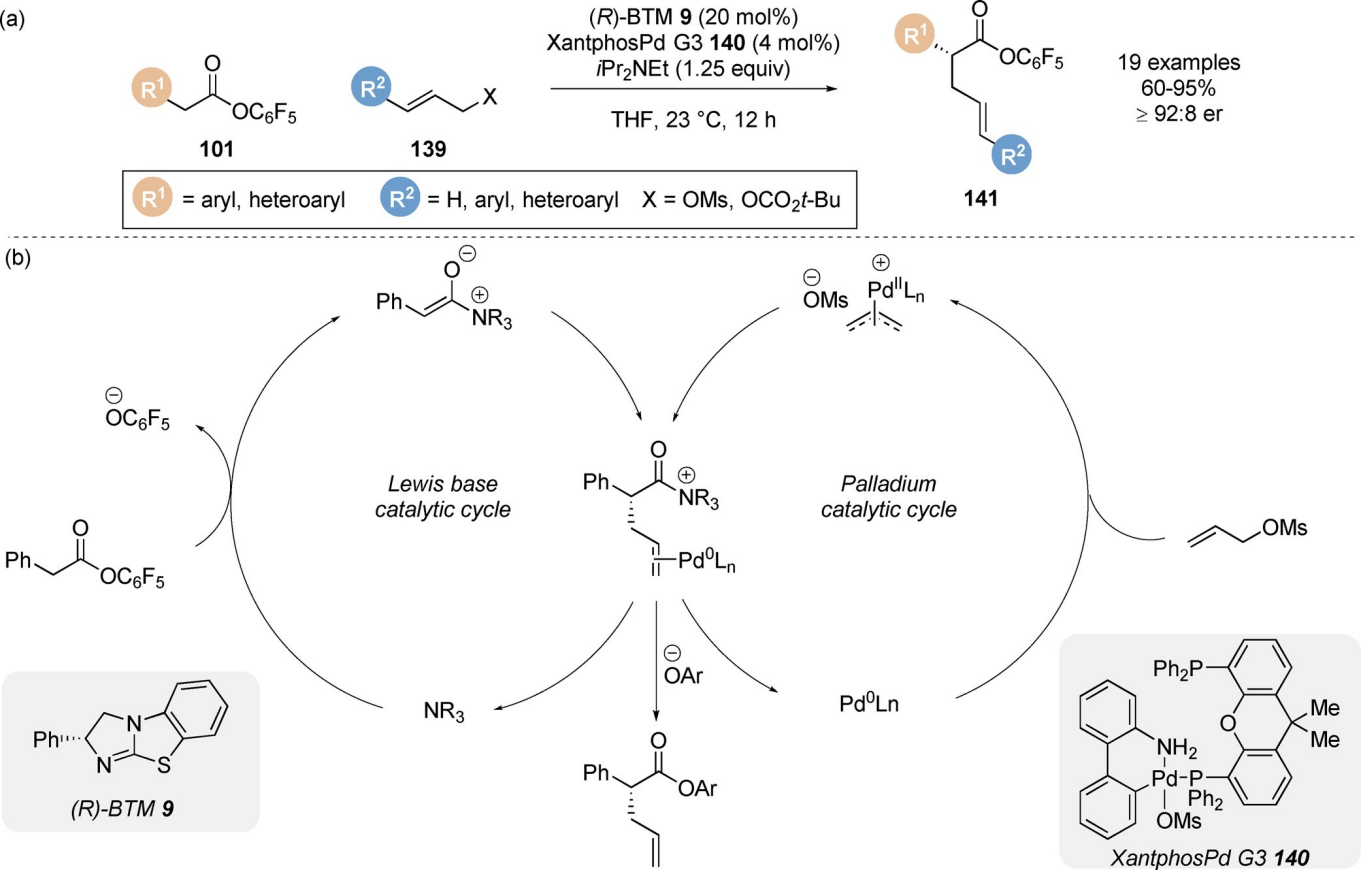
(a) Coorperative palladium/isothiourea catalysis for the enantioselective α‐allylation of pentafluorophenyl esters and (b) proposed mechanism.

Snaddon and co‐workers applied the aryloxide catalyst turnover strategy for the enantioselective α‐allylation of esters enabled by dual catalysis.^[56]^ In the presence of both (*R*)‐benzotetramisole **9** and XantphosPd G3 **140**, a range of pentafluorophenyl esters **101** underwent enantioselective α‐allylation with various allylic electrophiles **139** to give the corresponding linear α‐functionalised ester products **141** with excellent enantiocontrol (Scheme 30 a). The nature of the allylic leaving group had a marked effect on reactivity and enantioselectivity; allylic esters and chlorides gave the allylation products with poor enantioselectivity, whilst mesylate and *tert*‐butylcarbonate leaving groups gave the products in high yield and er. Pentafluorophenyl esters were found to be optimal for this dual‐catalytic system, allowing the products to be isolated in higher yield (due to increased chromatographic stability) and in shorter reaction times compared to other electron deficient aryl esters. The reaction mechanism for this process is proposed to involve the union of the C(1)‐ammonium enolate intermediate from the Lewis base nucleophilic catalytic cycle (left) and the π‐(allyl)Pd^II^ electrophile generated from the palladium cycle (right, Scheme 30 b). Critical to the success of merging these catalytic cycles is the reagent compatibility of each process; variation of either allylic nucleofuge, palladium catalyst, Lewis base or electron deficient aryl ester has a substantial effect on the reaction outcome.

**Scheme 31:**
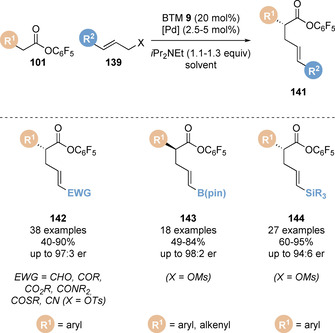
Palladium/isothiourea‐catalysed enantioselective α‐allylation using electron‐deficient, B(pin)‐substituted and silicon‐substituted allylic partners.

Building on this precedent, Snaddon and co‐workers have broadened the scope of this methodology to reactions with a range of different electrophilic partners bearing useful functional handles through modification of the palladium catalyst system (Scheme 31). Using the same Lewis base isothiourea catalyst, the regioselective addition of C(1)‐ammonium enolates, generated from pentafluorophenyl esters **101**, to electron deficient allylic tosylate partners **139** such as α,β‐unsaturated aldehydes, ketones, esters and amides has been achieved.^[73]^ Interestingly, a single tris[tri(2‐thienyl)phosphino]Pd^0^ catalyst system was found to be broadly applicable for each carbonyl functionality, despite discrepancies in electron‐withdrawing character, dipole and steric demand. Preliminary ligand studies indicated that the strong π‐accepting character of the ligand facilitates the preference for *syn* π‐(allyl)Pd^II^ intermediates, leading to regioselective (*E*)‐alkene isomer formation.

Employing Pd_2_dba_3_ and bidentate (*S*)‐BINAP catalyst‐ligand system in combination with (*S*)‐BTM was found to be optimal for the addition of enolates to B(pin)‐substituted allylic mesylate electrophiles.^[74]^ Notably, mismatched effects were observed when (*R*)‐BINAP was used with (*S*)‐BTM, where the enantioselectivity slightly decreased in this case. Aryl chlorides were tolerated in this process and did not undergo any competing Suzuki‐Miyaura cross‐coupling, whilst unproductive protodeboronation was also limited. B(MIDA) substituted allylic electrophiles were also demonstrated to be suitable in this process. The utility of the B(pin) substituent was demonstrated in a range of product derivatisations such as transesterification, C−O cross‐coupling and Suzuki‐Miyaura transformations. A Pd_2_dba_3_/P(2‐furyl)_3_ catalyst system was shown to enable the addition of C(1)‐ammonium enolates to silicon substituted allylic mesylate partners to afford the linear ester products **144**.^[75]^ The P(2‐furyl)_3_ ligand was found to suppress competing addition of pentafluorophenolate to the allylic electrophile partner, which was observed to limit the yield when other phosphine ligands were employed. The vinyl‐silane functionality contained in the products was utilised in an array of derivatisations such as halogenation, Hiyama–Denmark cross‐coupling and oxidation to the corresponding aldehyde.

**Scheme 32:**
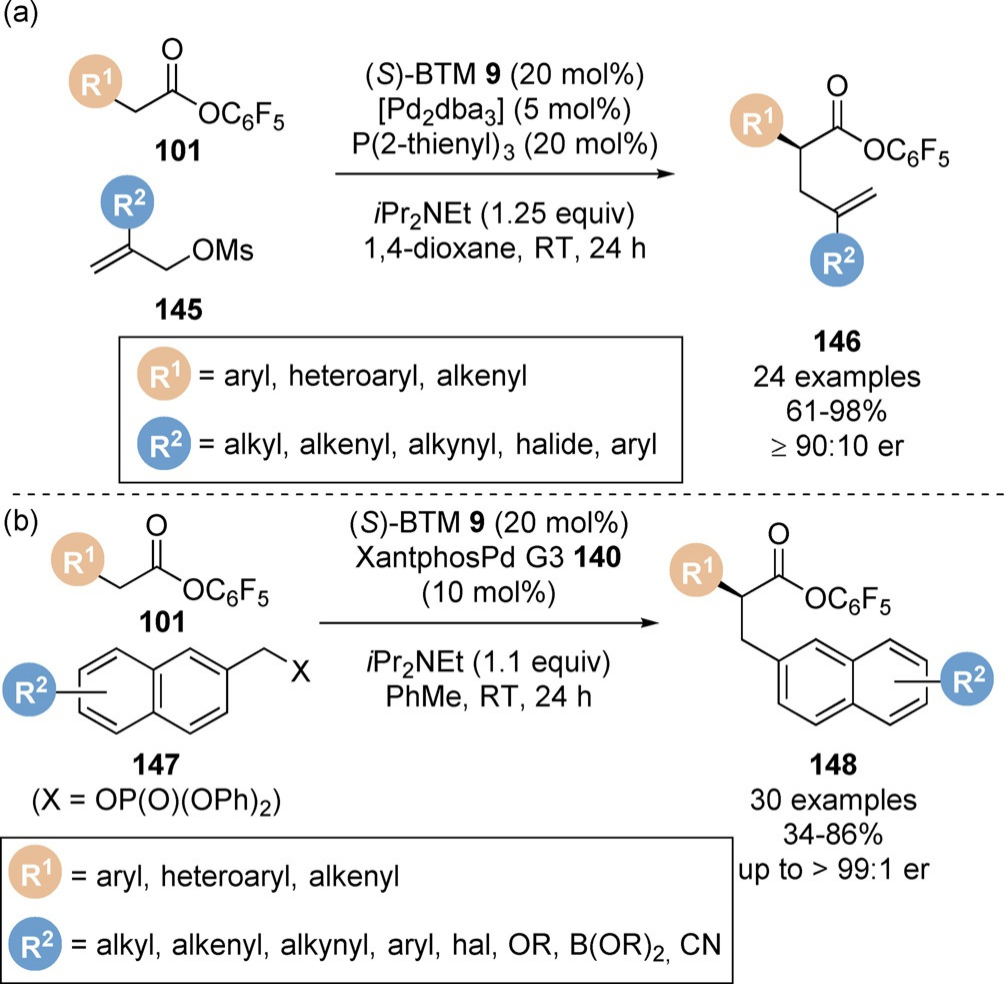
Dual palladium/isothiourea‐catalysed (a) enantioselective α‐allylation using 2‐substituted allyl electrophiles and (b) enantioselective α‐benzylation.

In each previous case the linear allylated products were observed. Snaddon and co‐workers postulated modulation of the palladium catalyst‐ligand system from a bidentate Xantphos ligand to a monodentate phosphine ligand would relieve steric congestion around the metal centre and engage 2‐substituted allyl partners. Indeed, a range of 2‐substituted allylic mesylates **145** underwent reaction with the corresponding pentafluorophenyl esters **101** using a monodentate, sterically undemanding 2‐thienyl phosphine ligand to give a range of branched α‐allylated esters **146** (Scheme 32 a).^[76]^ The product esters could be isolated, or derivatised in situ by addition of an appropriate nucleophile (amine or hydride) at the end of the reaction to give the corresponding amide or primary alcohol product. DFT studies were carried out to determine the nature of the transition state. This revealed a relatively low barrier (12.2 kcal mol^−1^) for an outer‐sphere attack of the enolate onto the π(allyl)Pd complex, whereas an inner‐sphere attack of a palladium‐ligated (*Z*)‐enolate was found to be highly disfavoured (29.8 kcal mol^−1^).

Snaddon and co‐workers later extended the scope of this methodology to benzylic electrophiles.^[77]^ Traditionally these are more challenging reaction partners due to the high energy of oxidative addition which requires dearomatisation of the arene unit.^[78]^ Critical to the success of this protocol was the identity of the nucleofuge (X): screening benzylic leaving groups revealed tosylate, acetate, *tert*‐butylcarbonate were all unreactive, with only diphenylphosphate proving productive. A range of benzylic phosphates **147** underwent reaction with pentafluorophenyl esters **101** in toluene catalysed by benzotetramisole **9** and Xantphos palladium **140** (Scheme 32 b). Notably a wide range of functional groups are tolerated under the reaction conditions including bromide, alkyne and boron‐containing functionality. The utility of this methodology was showcased in the synthesis of thrombin inhibitor DX‐9065A. However, the electrophilic partner was limited to π‐extended naphthyl groups. Monocyclic benzene‐derived electrophiles were unreactive presumably due to higher dearomatisation energy.

**Scheme 33:**
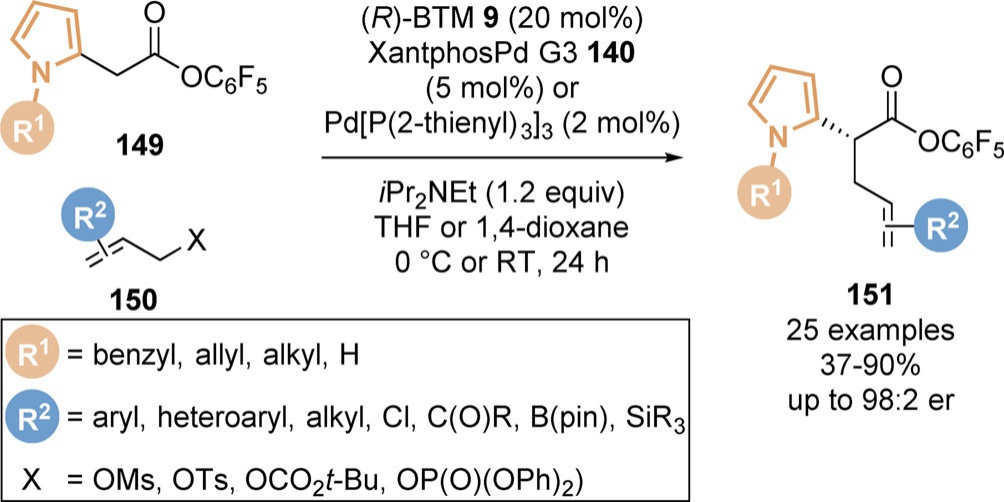
Dual palladium/isothiourea‐catalysed enantioselective α‐allylation of 2‐pyrrole substituted pentafluorophenyl esters.

Snaddon and co‐workers have also broadened the scope of the nucleophile component in the dual catalysed allylation protocol to pyrrole 2‐acetic acid pentafluorophenyl esters **149**, enabling the enantioselective synthesis of α‐alkylated pyrroles **151** (Scheme 33).^[79]^ *N*‐Alkyl, benzyl and allyl substituted pyrroles were tolerated, giving the corresponding allylated products in high yields with excellent enantioselectivity. Notably, no undesired allylation of the electron rich pyrrole ring was observed. The analogous unsubstituted pyrrole derivative also gave the desired product with useful enantioselectivity. Various allylic electrophile partners, in combination with the appropriate palladium catalyst system, could be employed for the synthesis of both linear and branched allylated products, with excellent functional group tolerance demonstrated (Cl, BPin, SiR_3_). The synthetic utility of the pyrrole products was also highlighted through a two‐step transesterification/ring closing metathesis sequence to form a bicycle pyrrole unit.

**Scheme 34:**
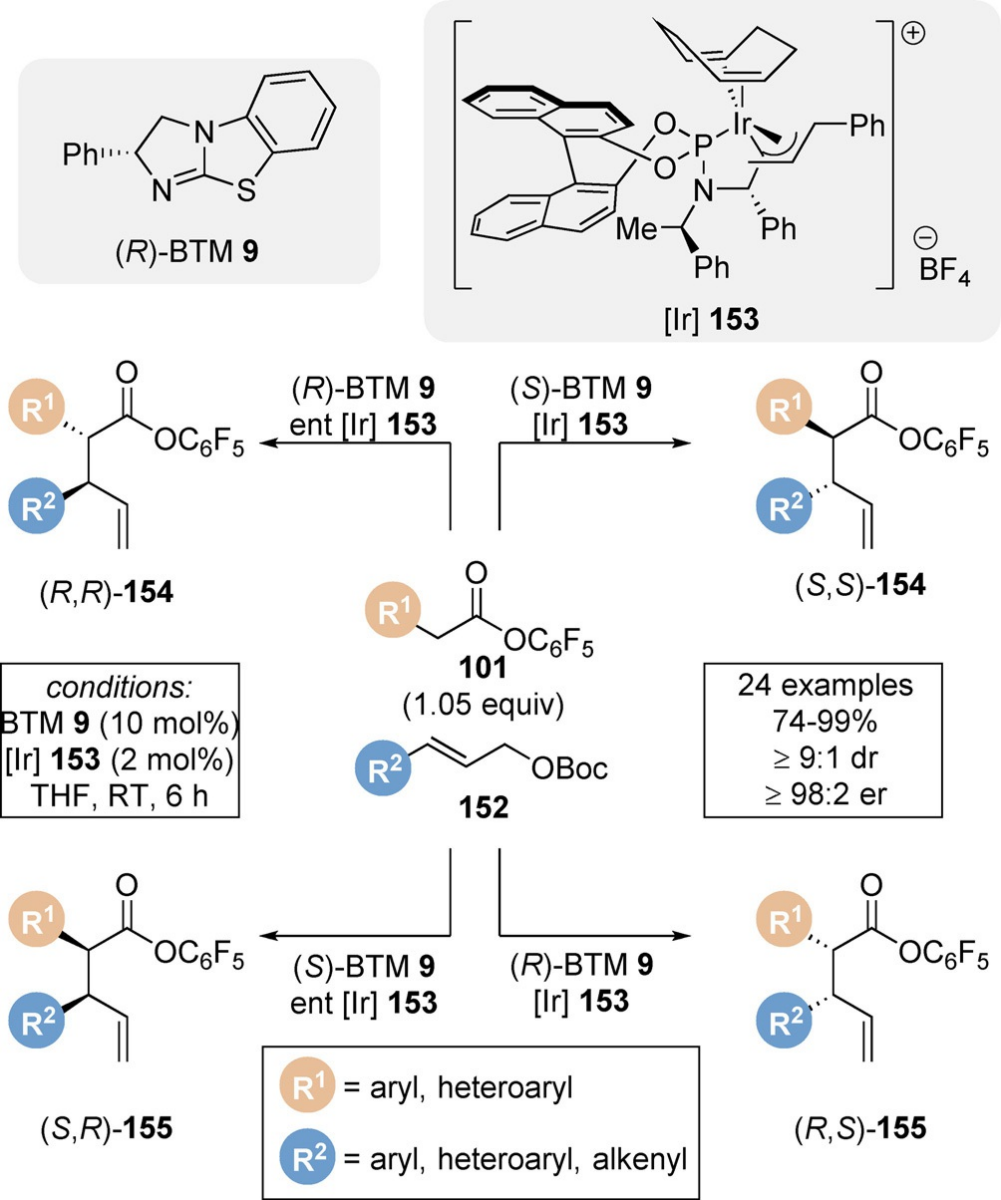
Dual iridium/isothiourea‐catalysed stereodivergent α‐allylation of pentafluorophenyl esters.

While Snaddon and co‐workers have varied the palladium catalyst to address reactivity challenges, Hartwig and co‐workers sought to access complementary reactivity through use of an alternative metal catalyst. The authors reported a related dual catalytic protocol using cooperative isothiourea/iridium catalysis, enabling exclusive formation of the branched allylated product. The enantioselective α‐allylation of pentafluorophenyl esters **101** was achieved giving branched products **154** and **155** (Scheme 34).^[80]^ This stereodivergent protocol allows access to all four product stereoisomers through predictable pairing of chiral catalyst enantiomers. Using each of the four different catalyst enantiomer combinations, each product stereoisomer could be isolated in high yield and in excellent dr and er, exemplifying the high control each catalyst exhibits over the substrates. Benzotetramisole **9** governs the absolute configuration of the C(2)‐carbon, whilst the metallacyclic iridium complex [Ir] **153** determines the geometry, facial selectivity and regioselectivity of the allyl electrophile, and therefore the absolute configuration at C(3). Notably, in this case the reaction conditions remain almost identical to the protocol developed by Snaddon and co‐workers. Through simple variation of only the metal catalyst, alternative reactivity has been achieved, highlighting the complementarity and potential of this synergistic methodology. A similar observation is also noticeable when comparing Gong and co‐workers carbonylation strategy (Scheme 24).

**Scheme 35:**
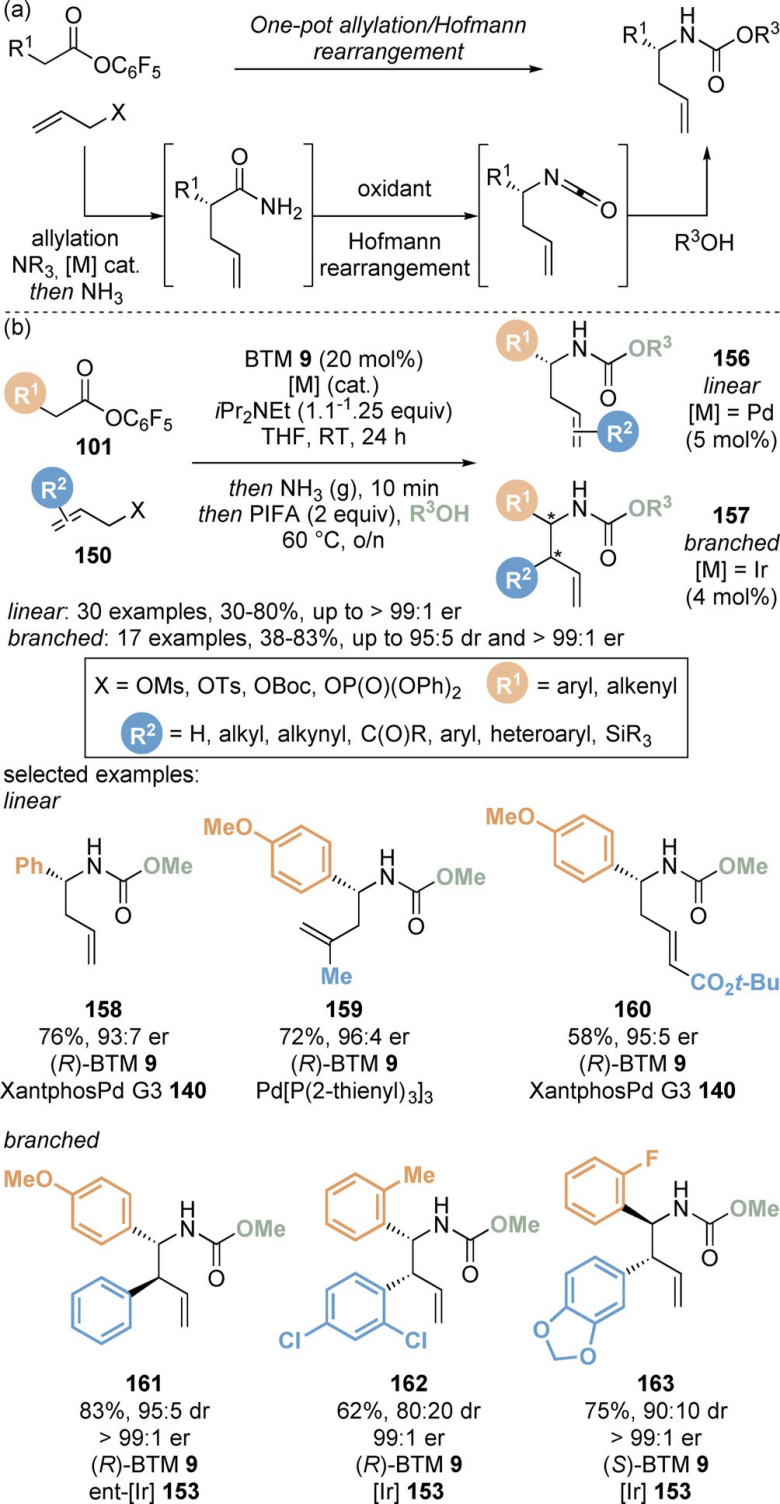
Enantioselective synthesis of homoallylic amines via a one‐pot allylation/Hofmann rearrangement sequence.

In 2019, Snaddon and co‐workers sought to pair the cooperative isothiourea/metal catalysis protocol with a Hofmann rearrangement^[81]^ for the one‐pot enantioselective synthesis of homoallylic amines.^[82]^ It was proposed in situ conversion of the allylated ester products to the primary amide could be achieved using ammonia gas. Subsequent treatment with an oxidant would enable stereospecific Hofmann rearrangement to give the corresponding isocyanate, which could be quenched with an appropriate alcohol forming the carbamate‐protected homoallylic amines (Scheme 35 a). A range of pentafluorophenyl esters **101** and allylic electrophiles **150** successfully underwent the one‐pot allylation‐amidation‐rearrangement procedure through treatment with ammonia gas at the end of the allylation reaction, followed by oxidation using [bis(trifluoroacetoxy)iodo]benzene (PIFA). Both the linear (**156**) and branched (**157**) products could be conveniently accessed in good yield with high enantioselectivities through simple choice of palladium or iridium catalysts, respectively (Scheme 35 b). This regio‐ and stereodivergent one‐pot approach allows convenient and modular access to a variety of enantioenriched homoallylic amines. Notably, simple variation of the alcohol allows various protected amines to be synthesised, and the addition of water allows formation of the free amine.

### 3.3 α‐Functionalisation of esters: Alternative electrophiles

**Scheme 36:**
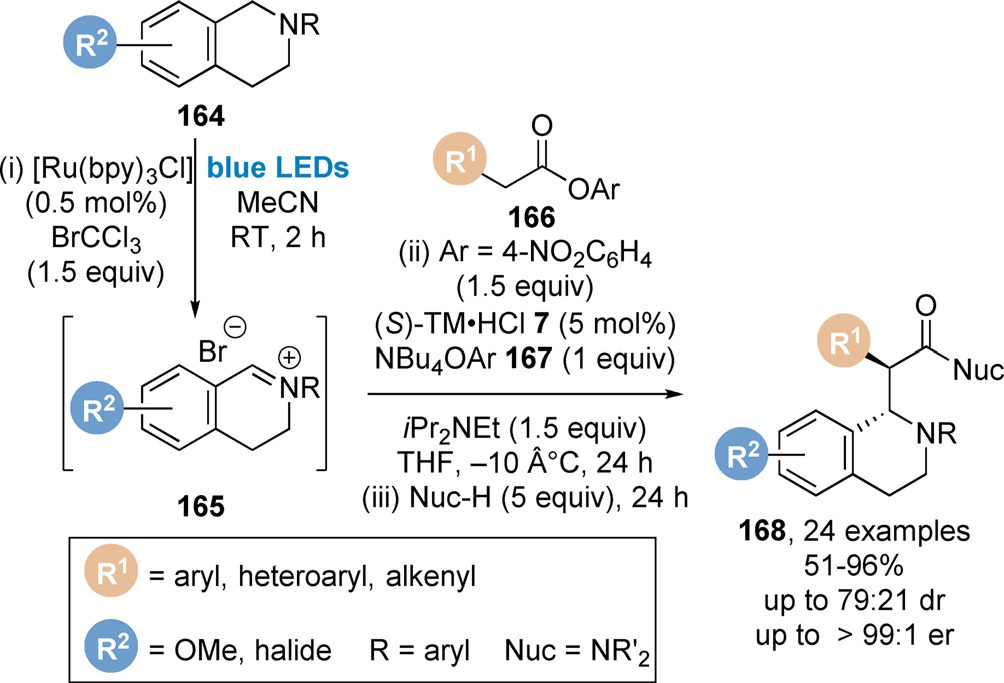
Enantioselective addition of C(1)‐ammonium enolates, generated from *para*‐nitrophenyl esters to iminium ion electrophiles.

In 2018, using *para*‐nitrophenyl esters **166** as C(1)‐ammonium enolate precursors, Smith and co‐workers reported the isothiourea‐catalysed addition to tetrahydroisoquinoline‐derived iminium ions **165** (Scheme 36).^[83]^ An appropriate amine nucleophile was added at the end of the catalytic reaction to convert the less stable *para*‐nitrophenyl ester product to the corresponding amide. During the optimisation of this process addition of stoichiometric tetrabutylammonium *para*‐nitrophenoxide **167** was shown to give increased yields and enantioselectivities. This is proposed to increase polarity of the reaction mixture whilst also accelerating the rate of catalyst turnover. Also noteworthy was the effect that the iminium counterion had on product enantioselectivity. Small, coordinating halides (Br^−^, Cl^−^) gave higher enantioselectivities than larger, non‐coordinating anions such as BF_4_^−^, PF_6_^−^ and BPh_4_^−^. The iminium bromide ions **165** could also be generated in situ via photoredox catalysis using BrCCl_3_ and Ru(bpy)_3_Cl_2_, allowing for the development of a one‐pot sequential strategy. Using this sequential photoredox/Lewis base‐catalysed procedure, a range of *para*‐nitrophenyl esters **166** could be converted to the β‐amino amide products **168** in good yield with high enantioselectivity, but with low diastereoselectivity (=75:25 dr). This process overcomes some of the challenges associated with aryloxide catalyst turnover such as compatibility of nucleophilic (aryloxide, Lewis base catalyst) and electrophilic species (iminium ion) within the reaction mixture.

These impressive methodologies using aryloxide catalyst turnover have significantly broadened the reaction scope, showcasing the reaction of C(1)‐ammonium enolate intermediates in dual‐catalytic processes and with alternative electrophiles for the enantioselective α‐functionalisation of acyclic esters. However, in these previous processes there is a typical requirement for relatively high catalyst loadings (often 20 mol %) and/or excess auxiliary Brønsted base (necessary to neutralise acidic by‐products) for effective reactivity. In addition, these processes had not been investigated to identify reaction intermediates, orders with respect to each component and turnover limiting steps.

**Scheme 37:**
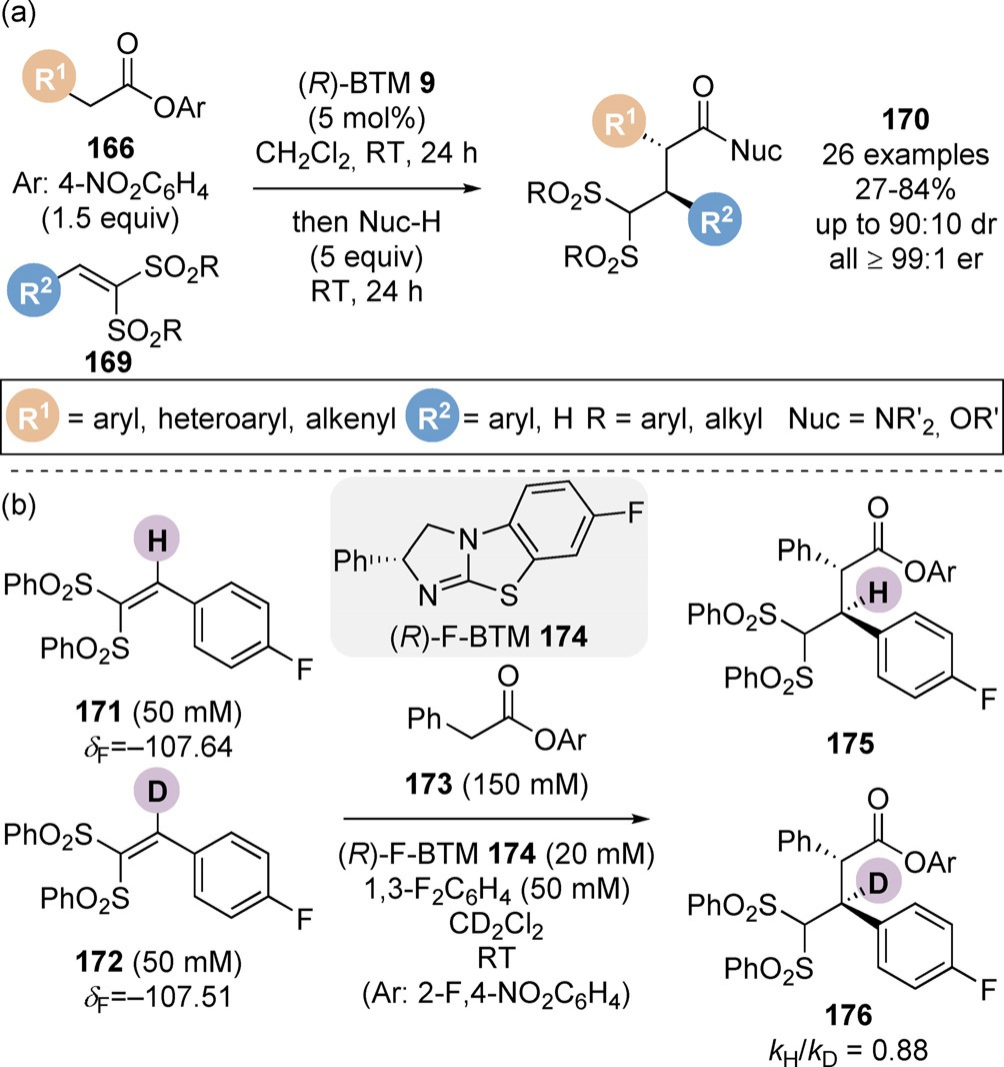
(a) Base‐free enantioselective C(1)‐ammonium enolate catalysis and (b) observed inverse secondary kinetic isotope effect.

In 2019, Smith and co‐workers developed a base‐free protocol for the enantioselective α‐functionalisation of esters (Scheme 37 a).^[84]^ Reaction of a range of *para*‐nitrophenyl esters **166** with vinyl bis‐sulfone Michael acceptors **169** catalysed by only 5 mol % (*R*)‐benzotetramisole **9** gave α‐alkylated *para*‐nitrophenyl ester and amide products **170** (after derivatisation) in excellent yield and stereoselectivity. Key to the success of this methodology was the multifunctional aryloxide which operates as a leaving group, Brønsted base, Brønsted acid (as the corresponding phenol) and as a Lewis base within the catalytic cycle, allowing for the reaction to be carried out in the absence of an auxiliary base. The steric and electronic nature of the aryloxide was assessed. It was concluded that esters derived from phenols with a lower p*K*_a_ value (pentafluorophenyl, tetrafluorophenyl)^[85]^ gave decreased reactivity, whilst esters of higher p*K*_a_‐ such as the ester derived from phenol‐ were completely unreactive. The ester derived from *para*‐nitrophenol, whose p*K*_a_ lies between these two extremes,^[86]^ gave optimal yields. This is proposed to be due to a careful balance of leaving group ability, amphoteric behaviour and steric effects,^[87]^ where *para*‐nitrophenol is the most effective aryloxide for this application. The reaction was also demonstrated to be functional in environmentally benign solvents such as dimethylcarbonate, isopropyl acetate and 2‐ methyl THF.^[88]^ The functional products could be deprotected upon treatment with magnesium turnings to form α‐alkylated amides without loss of enantiopurity. The pronucleophilic nature of the sulfone functional handle was also exploited under basic conditions in the presence of benzyl bromide or methyl vinyl ketone to provide chain extended amides.

Comprehensive mechanistic investigations were carried out to interrogate the reaction mechanism. Quantitative ^19^F{^1^H} NMR was employed for reaction monitoring and to identify the catalyst resting state (free catalyst) and deactivation through protonation. When employed in tandem with variable time normalisation analysis (VTNA)^[41]^ the reaction orders with respect to each component were determined. The ester, vinyl bis‐sulfone and catalyst were all determined to be first order, indicating the turnover limiting step involved each component, and was either Michael addition or catalyst turnover. A competition reaction between proto‐vinyl bis‐sulfone **171** and deutero‐vinyl bis‐sulfone **172** revealed an inverse secondary kinetic isotope effect (Scheme 37 b), confirming the Michael addition as the turnover limiting step. Product inhibition was also observed during the mechanistic analysis. It is proposed the acidic proton within the product (adjacent to sulfonyl groups) may inhibit the reaction by protonating either the C(1)‐ammonium enolate or aryloxide, thus retarding the rate of catalyst turnover.

**Scheme 38:**
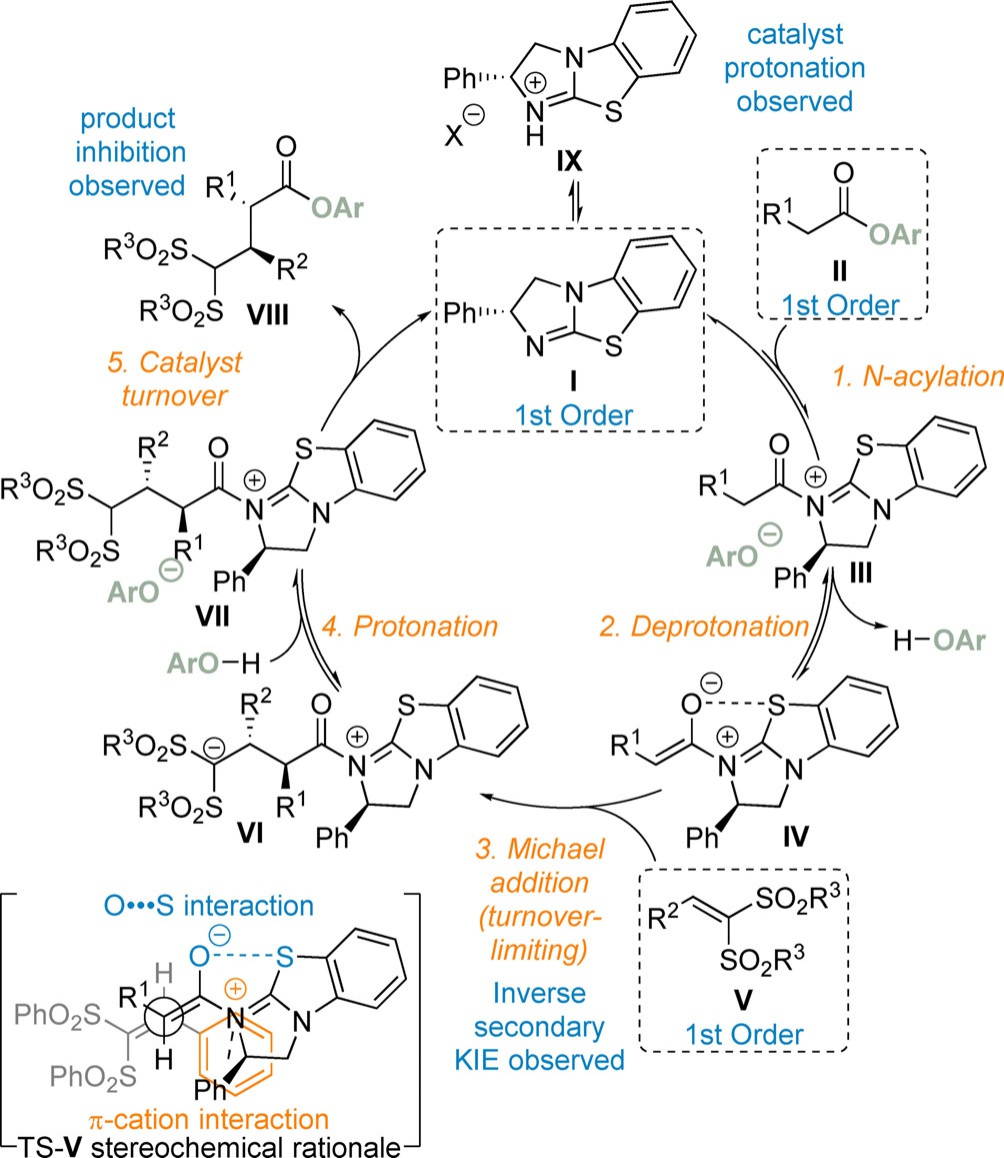
Proposed mechanism of base‐free enantioselective C(1)‐ammonium enolate catalysis featuring a multifunctional aryloxide.

The reaction mechanism was proposed to proceed by reversible *N*‐acylation of benzotetramisole catalyst **I** with ester **II** to form acyl ammonium ion pair **III** (Scheme 38). Reversible deprotonation of the acyl ammonium ion by the aryloxide counter anion affords the nucleophilic C(1)‐ammonium enolate **IV** and releases *para*‐nitrophenol. Turnover limiting Michael addition to the electrophile **V** on the *Re* face of the enolate leads to intermediate **VI**. Protonation by the *para*‐nitrophenol released in step two gives acyl ammonium ion pair **VII**. Addition of the aryloxide anion forms the product **VIII** and regenerates catalyst **I**, which is in equilibrium with the catalytically inactive protonated‐BTM **IX**. The observed diastereoselectivity can be rationalised tentatively by a favoured open pre‐transition state assembly TS‐**V** where gauche interactions are minimised about the forming C−C bond while allowing a potentially favourable π‐cation interaction between the β‐substituent of the bis‐sulfone electrophile and the isothiouronium cation.^[68]^ The relative configuration within the major diastereoisomer obtained using these bis‐sulfone electrophiles is opposite to that observed in previous isothiourea catalysis employing intramolecular catalyst turnover processes and in the intermolecular addition to iminium ions. This difference can presumably be rationalised due to the two highly sterically demanding sulfone groups of this series of electrophile. Whilst this process enables the generation of a C(1)‐ammonium enolate without the requirement for use of an excess auxiliary base, a clear limitation is the use of a highly reactive vinyl bis‐sulfone Michael acceptors. Electrophiles bearing only one sulfonyl electron‐withdrawing group were unreactive in this protocol. Therefore, a more general solution remains elusive.

**Scheme 39:**
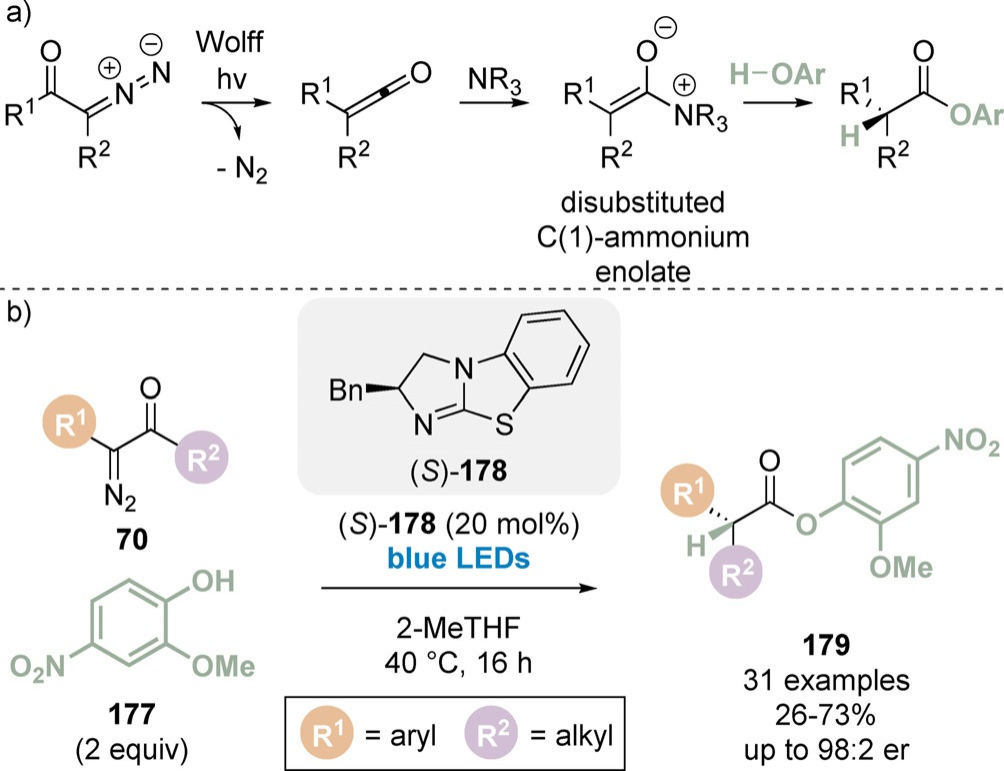
Enantioselective protonation of a disubstituted C(1)‐ammonium enolate generated from α‐dizaoketones.

Employing a similar strategy to Song and co‐workers,^[45]^ Han and co‐workers reported the enantioselective protonation of C(1)‐ammonium enolates generated from α‐diazoketones through a visible‐light‐induced ketene formation (Scheme 39).^[89]^ The transformation is proposed to proceed through Wolff rearrangement of α‐diazoketones to give disubstituted ketenes, which can be intercepted by a Lewis base to give the disubstituted C(1)‐ammonium enolate. Protonation and catalyst turnover by a corresponding phenol gives access to α,α‐disubstituted carboxylic esters (Scheme 39 a). A range of α‐aryl‐α‐diazoalkylketones **70** smoothly underwent the rearrangement/enantioselective protonation sequence when treated with phenol **177** and isothiourea catalyst **178** under blue LED irradiation to give the corresponding α‐alkyl‐α‐aryl‐ester products **179** (Scheme 39 b). The method was also used to prepare (*R*)‐ibuprofen in two steps from the corresponding α‐diazoketones and phenol **177**. Substitution in the 2‐position with an electron‐donating group, and an electron‐withdrawing group in the 4‐position of the phenol is required for high enantioselectivities.

## Conclusions and Outlook

C(1)‐Ammonium enolates are effective and versatile synthetic intermediates for enantioselective α‐functionalisation of carbonyl units at the carboxylic acid oxidation level. In recent years, new methods for generating C(1)‐ammonium enolates from different precursors have been developed using acyl imidazoles, electron deficient aryl esters and α‐diazoketones. Through catalyst turnover by lactonisation/lactamisation, many enantioenriched chiral heterocycles can be prepared efficiently. Enantioselective α‐functionalisation of acyclic esters has been recently achieved by employing electron deficient aryl esters as enolate precursors. Advances in catalyst design have also been achieved through the application of immobilised catalysts and isoselenoureas. Despite these advances, many opportunities remain for the development of novel reactions, particularly with aryloxide catalyst turnover which has been less explored but is an expanding research area. Application for the synthesis of complex target molecules and use in industry is desirable, especially owing to the mild reaction conditions and functional group tolerance. Reactivity limitations remain, such as the general need for an aryl or alkenyl substituent in the α‐position. In addition, access to disubstituted enolates for the synthesis of quaternary centres remains limited. The majority of examples involve the addition to sp^2^‐hybridised carbon electrophiles. Whilst Lectka has demonstrated C−X bonds can be formed via C(1)‐ammonium enolate intermediates, this process uses acid chloride/ketene starting materials. Expansion of compatible electrophiles in combination with aryloxide catalyst turnover using aryl ester precursors to form C−X bonds would also be clearly beneficial. Finally, a continued advancement to more sustainable, atom‐economical processes using environmentally friendly solvents would make the area more appealing for industrialists.

## Conflict of interest

The authors declare no conflict of interest.

## Biographical Information

*Calum McLaughlin obtained a Masters degree from the University of Strathclyde (2016) supervised by Dr. Allan Watson. During this time*, *he undertook an industrial placement year at GSK (Stevenage) and research placements with Dr. Allan Watson*, *Prof. William Kerr and Prof. Eva Hevia. He is currently a final year PhD student at the University of St Andrews in the group of Prof. Andy Smith*, *researching enantioselective isothiourea catalysis*.

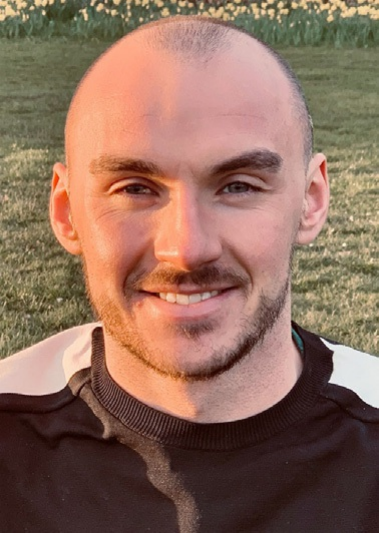

## Biographical Information

*Andy Smith was appointed at St. Andrews in October 2005 and promoted to Professor in 2012. He was awarded the RSC Merck Award in 2014 and the RSC Charles Rees Award in 2018. His research programme is focused on catalytic enantioselective reaction processes using Lewis base catalysts and developing a comprehensive mechanistic understanding of these transformations*.